# Epstein-Barr virus nuclear antigen EBNA-LP is essential for transforming naïve B cells, and facilitates recruitment of transcription factors to the viral genome

**DOI:** 10.1371/journal.ppat.1006890

**Published:** 2018-02-20

**Authors:** Agnieszka Szymula, Richard D. Palermo, Amr Bayoumy, Ian J. Groves, Mohammed Ba abdullah, Beth Holder, Robert E. White

**Affiliations:** 1 Section of Virology, Department of Medicine, Imperial College London, London, United Kingdom; 2 Section of Pediatrics, Department of Medicine, Imperial College London, London, United Kingdom; Tulane Health Sciences Center, UNITED STATES

## Abstract

The Epstein-Barr virus (EBV) nuclear antigen leader protein (EBNA-LP) is the first viral latency-associated protein produced after EBV infection of resting B cells. Its role in B cell transformation is poorly defined, but it has been reported to enhance gene activation by the EBV protein EBNA2 in vitro. We generated EBNA-LP knockout (LPKO) EBVs containing a STOP codon within each repeat unit of internal repeat 1 (IR1). EBNA-LP-mutant EBVs established lymphoblastoid cell lines (LCLs) from adult B cells at reduced efficiency, but not from umbilical cord B cells, which died approximately two weeks after infection. Adult B cells only established EBNA-LP-null LCLs with a memory (CD27+) phenotype. Quantitative PCR analysis of virus gene expression after infection identified both an altered ratio of the EBNA genes, and a dramatic reduction in transcript levels of both EBNA2-regulated virus genes (LMP1 and LMP2) and the EBNA2-independent EBER genes in the first 2 weeks. By 30 days post infection, LPKO transcription was the same as wild-type EBV. In contrast, EBNA2-regulated cellular genes were induced efficiently by LPKO viruses. Chromatin immunoprecipitation revealed that EBNA2 and the host transcription factors EBF1 and RBPJ were delayed in their recruitment to all viral latency promoters tested, whereas these same factors were recruited efficiently to several host genes, which exhibited increased EBNA2 recruitment. We conclude that EBNA-LP does not simply co-operate with EBNA2 in activating gene transcription, but rather facilitates the recruitment of several transcription factors to the viral genome, to enable transcription of virus latency genes. Additionally, our findings suggest that EBNA-LP is essential for the survival of EBV-infected naïve B cells.

## Author summary

Epstein-Barr virus (EBV) infects almost everyone. Once infected, people harbor the virus for life, shedding it in saliva. Infection of children is asymptomatic, but a first infection during adolescence or adulthood can cause glandular fever (infectious mononucleosis). EBV is also implicated in several different cancers. EBV infection of B cells (antibody-producing immune cells) can drive them to replicate almost indefinitely (‘transformation’), generating cell lines. We have investigated the role of an EBV protein (EBNA-LP) which is thought to support gene activation by the essential virus protein EBNA2. We have made an EBV in which the *EBNA-LP* gene has been disrupted. This virus (LPKO) shows several properties. 1. It is reduced in its ability to transform B cells; 2. ‘Naïve’ B cells (those whose antibodies have not adapted to fight infections) die two weeks after LPKO infection; 3. Some virus genes fail to turn on immediately after LPKO infection. 4. Binding of EBNA2 and various cellular factors to these genes is delayed. 5. EBNA-LP does not affect EBNA2-targeted cellular genes in the same way. This shows that EBNA-LP is more important in naïve B cells, and that it helps to turn on virus genes, but not cell genes.

## Introduction

Epstein-Barr virus is a ubiquitous human herpesvirus that asymptomatically infects the vast majority of the human population, particularly in the developing world, where primary infection typically occurs during the first few years of life, leading to lifelong EBV latency. Where primary infection is delayed into adolescence or adulthood, it can result in the temporarily debilitating but relatively benign condition, infectious mononucleosis. The major disease burden caused by EBV is the range of malignancies with which it has been associated. In particular EBV contributes to high levels of Burkitt lymphoma in sub-Saharan Africa and of nasopharyngeal carcinoma in southeast Asia, as well as around a third of Hodgkin lymphoma cases, approximately one in ten gastric cancers, a range of B cell lymphomas in the immunosuppressed and more rarely with T and NK cell malignancies. Taken together EBV is implicated in around 1-1.5% of worldwide cancer incidence [1].

These diverse malignancies likely arise due to defects at different stages of the virus life cycle, or perhaps infection of cell types not involved in the virus’s natural life cycle [2]. The core of the EBV lifecycle occurs with the B cell compartment. EBV infection activates B cells, transforming them into proliferating lymphoblasts. In vitro these continue to proliferate into lymphoblastoid cell lines (LCLs) whereas in vivo they can differentiate – probably via a germinal center – into resting memory B cells where the virus is quiescent, producing RNAs but no viral proteins [3,4]. LCLs express the ‘growth’ program of EBV genes (latency state III), comprising six EBV nuclear antigens (EBNAs), the latency membrane proteins (LMP1, LMP2A and LMP2B) and a number of EBV encoded RNAs, including the abundant nuclear RNAs EBER1 and EBER2.

The latency III transcriptional program takes over 2 weeks to reach this state [5]. The first EBV proteins detectable after primary infection are EBNA2 and the EBNA leader protein (EBNA-LP) [6,7]. Shortly after this the EBNA3 proteins also become detectable [6], and the EBNAs rapidly reach the levels found in LCLs. In contrast, the LMP proteins take up to three weeks to reach LCL-like levels [5] [8]. EBNA transcription is initiated at multiple copies of Wp, the promoter in the major internal repeat (IR1) of EBV. Soon after infection, the burden of EBNA transcription shifts to Cp, a promoter upstream of IR1. Transcripts from both Cp and Wp are alternatively spliced, and translated in both cap- and IRES-dependent manners to produce the six EBNAs.

The functions of most of the EBNAs are reasonably well understood: EBNA1 is important for the replication and segregation of the viral genome during the cell cycle, by binding to oriP. The EBNA1/oriP complex is also important in the switch from Wp to Cp [9]. EBNA2 is essential for the initial transformation of B cells, rapidly activating both host and viral genes through its recruitment to promoters or enhancers alongside cellular transcription factors such as Pu.1 [10], RBPJ (also called CBF1) [11],[12], IRF4 and EBF1 [13,14]. The EBNA3 proteins are slow-acting transcriptional repressors that are important for suppressing senescence and apoptosis around 3 weeks after infection [15,16]. Notably, the EBNA3s and EBNA2 appear to have a close relationship with each other, co-regulating genes and being bound at many of the same chromosomal locations [13,17–19].

In contrast to the other EBNAs, the role played by EBNA-LP in B cell transformation is not clear. Through initiation at different Wp promoters and exon skipping in Cp transcripts, the EBNA-LP protein comprises a variable copy number of a 66 amino-acid N terminal repeat domain (encoded by exons W1 and W2 within IR1) and a C terminal domain encoded by exons Y1 and Y2. In LCLs, EBNA-LP mainly localizes to PML nuclear bodies [20] – nuclear foci implicated in repression of virus infection, also called nuclear domain 10 (ND10) [21] – although it takes several days after infection to accumulate there [22]. Functionally, EBNA-LP has been shown to enhance the activation of host and viral genes by EBNA2 after transfection, although not all studies agree on which genes are affected [7,23–27].

The complex repetitive nature of the *EBNA-LP* gene makes its analysis in the viral context challenging. Previous genetic analyses of EBNA-LP have been restricted to mutation of the C-terminal Y exons [28,29]. These Y domain knockout viruses establish LCLs at a much reduced efficiency, and then only when the early outgrowth of the cell lines was supported by growth on irradiated fibroblast feeder cells. Deleting IR1 repeat units below five progressively reduced transformation efficiency [30], but as well as reducing EBNA-LP size, this reduced transcript dosage of EBNA-LP and EBNA2 – and probably of the recently identified stable intronic sequence RNAs (sisRNA1 and sisRNA2) [31] – due to the reduced Wp number producing fewer EBNA transcripts.

These prior studies of EBNA-LP function have been conducted in the context of transfecting isolated genes, and/or in the presence of the truncated EBNA-LP protein produced by the P3HR1 virus, and not in the context of virus infection. Therefore, the aim of this project was to produce a complete EBNA-LP knockout virus, and use it to establish the importance of (and a role for) EBNA-LP in the transformation of B cells. While our first EBNA-LP knockout was additionally defective due to unintended mutations in the introns between the EBNA-LP exons in IR1, a second, cleaner knockout showed that EBNA-LP is important but dispensable for the transformation of adult memory B cells, but is essential for the transformation of naïve B cells. Furthermore, both knockouts demonstrated that EBNA-LP is crucial for establishing and stabilizing the viral transcription program after infection, and for facilitating the recruitment of EBNA2 and the host protein EBF1 to the incoming virus genome. However, EBNA-LP did not enhance the induction of host genes by EBNA2 during infection, which had been expected based on prior understanding of its function.

## Results

### Generation and validation of EBNA2- and EBNA-LP-deficient EBVs reveals a role for IR1 intronic sequences in transformation

**Fig 1:**
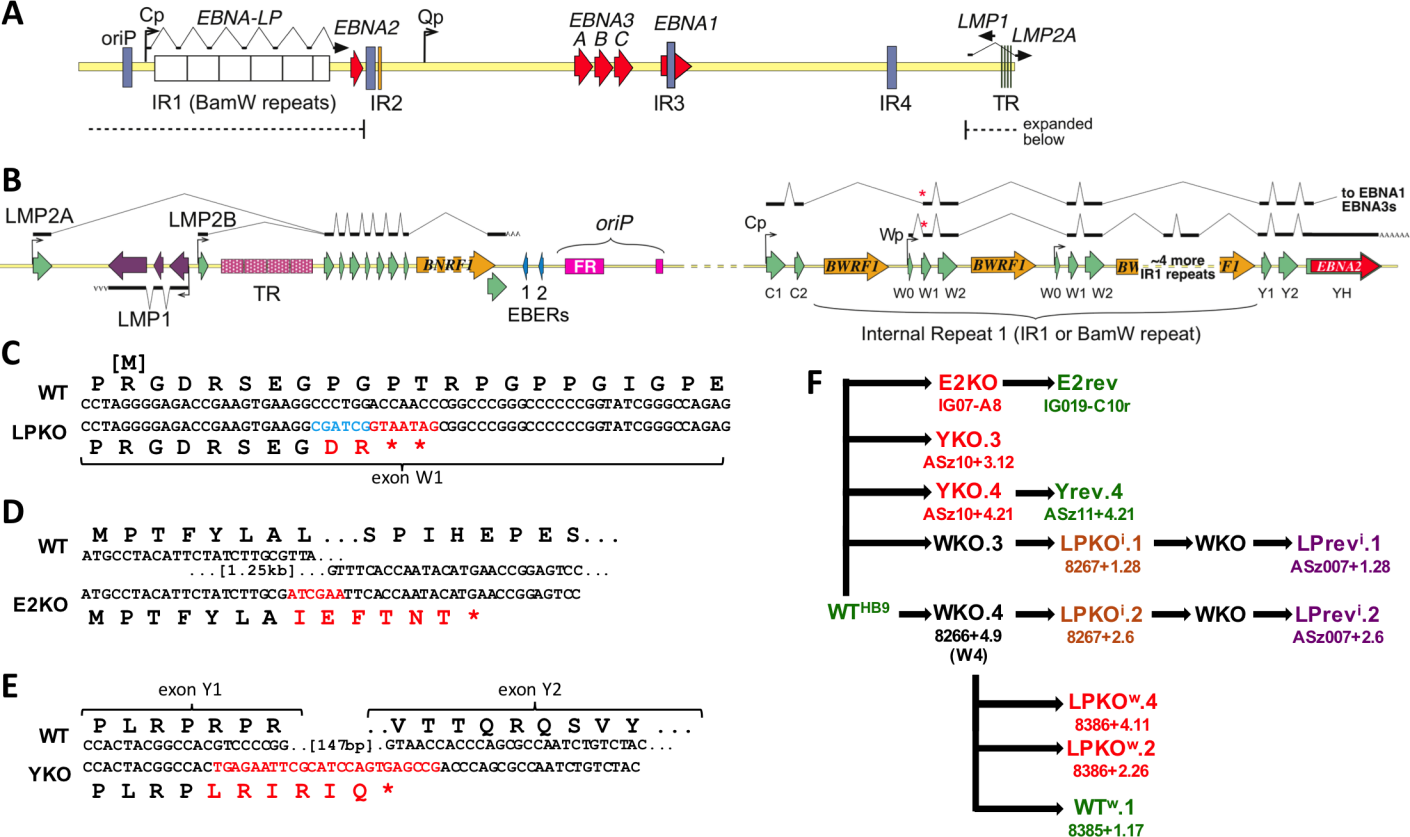
Construction of EBNA-LP knockouts and their revertants. **A.** Schematic representation of the EBV genome showing the latency genes and internal repeat 1 (IR1) across which the repetitive domain of EBNA-LP is transcribed. Region from the LMP genes to EBNA2 (dotted line) crosses the termini of the linear genome and is shown in **B.** Promoters Cp and Wp are indicated by bent arrows; exons are green (right facing) or purple (left facing) arrows, with intact ORFs indicated in other colours. Names of EBNA exons are shown below the relevant arrow. The splicing of latency genes is indicated above (plus strand transcripts) or below (minus strand transcripts) the exons. Two examples of the alternatively spliced EBNA transcripts are shown, indicating variable numbers of W exon pairs in EBNA-LP, variable use of Cp and Wp promoters, and differential splicing producing either EBNA2 or other downstream EBNAs. Variations in the W1 splice acceptor from C2 or W0 (red asterisks) define whether a transcript encodes EBNA2 or EBNA-LP. **C-E** Mutations introduced into recombinant viruses. Parental B95-8 sequence is shown above the introduced mutation used to generate: **C.** EBNA-LP knockout; **D.** EBNA2 knockout; **E.** EBNA-LP Y exon knockout. **F.** Flow chart showing set of recombineering steps used to generate the recombinant EBVs constructed for this study. Note that viruses whose IR1 is mutated are made via an intermediate (WKO) in which IR1 has been entirely deleted. Coloured names indicate recombinant BACs that were used to generate the viruses used in experiments–Green names are wild-type in sequence and phenotype; Red names are mutants; LPrev^i^ is shown in purple, as it contains a point change compared to wild-type that was intended to be phenotypically neutral. Alternative lab names are included for reference.

EBNA-LP is transcribed across IR1 (Fig 1A). Each repeat unit contains the Wp promoter from which its transcription can initiate, and repeat units can be skipped by splicing (Fig 1B). Therefore the only approach to reliably knockout EBNA-LP is to introduce a nonsense mutation into the *EBNA-LP* coding region in each of the IR1 repeat units of EBV. To do this, we generated an array of 6.6 IR1 repeat units (typical for circulating viruses [32], and matching the size in the parental B95-8 BAC, WT^HB9^) containing a STOP codon mutation (and restriction site for screening – Fig 1C).

In order to separate the role of EBNA2 from that of EBNA-LP, an EBNA2 knockout (E2KO) EBV – and its revertant, E2rev – were also generated. E2KO retains the entire Y3 exon and its 3’ splice site. This allows qPCR detection of Y2-YH EBNA2 transcripts in the E2KO, despite being deleted for the rest of the *EBNA2* ORF (Fig 1D). In order to facilitate comparison with the previous genetic studies of EBNA-LP function in a P3HR1 strain backbone [28,29], we also generated a pair of recombinant viruses (designated YKO) that deleted a genome region from within exon Y1 to within exon Y2. This deleted the Y exon encoded region of *EBNA-LP*, but retained exon Y1 splice acceptor and exon Y2 splice donor (Fig 1E). A revertant (Yrev) was generated for one of these knockouts.

Two distinct approaches were used to generate the IR1 repeat arrays used to generate LPKO viruses. The first used type IIS restriction enzymes, a method that necessitated the inclusion of a point mutation in the small intron between exons W1 and W2 (S1 Fig). Duplicate knockouts were made by this method, and a revertant was made of each (Fig 1F). Because of the intronic mutation, these viruses were designated LPKO^i^ and LPrev^i^ respectively [the ‘i' indicating intronic mutation]. The recombinant viruses were checked for unwanted deletions by restriction digest and pulsed field gel electrophoresis (S2 Fig). It was subsequently discovered that the IR1 repeat unit used to clone LPKO^i^ and LPrev^i^ viruses contained three minority variants (in the BWRF1 putative open reading frame), normally found in only one IR1 repeat unit of B95-8 [33]. Thus LPKO^i^ and LPrev^i^ have four nucleotide changes to the intronic sequences in IR1, relative to the B95-8 consensus sequence.

Recombinant virus genomes were transfected into 293 cells and clones selected from which virus could be produced. EBV-BAC DNA was rescued into bacteria from these producer cell lines to ensure that the virus genome was unchanged. The recombinant viruses were initially assessed in BL31 cells, which can be used to assess EBVs regardless of whether they are capable of transforming primary B cells into LCLs [34,35]. The mutations did not alter the splicing of EBNA transcripts initiated at either Cp or Wp (S3A Fig), other than the expected shortening of transcripts in YKO cell lines caused by the deletion in the Y1 and Y2 exons. Nor did they reduce the levels of any latency proteins other than those that had been mutated (S3B Fig, S4 Fig). EBNA-LP protein levels were higher in E2KO-infected BL31, while LPrev^i^ viruses tended to have both larger and more abundant EBNA-LP isoforms. In contrast EBNA-LP levels were very low in YKO-infected cells. These changes in EBNA-LP levels were also seen by immunofluorescence in newly infected primary B cells, which also showed that most of the truncated EBNA-LP in YKO cells was restricted to a nuclear subregion, possibly the nucleolus (S5 Fig). We have recently shown that the B95-8 strain of EBV, and the WT^HB9^ BAC contain a stop codon at the end of exon W1 in one of its IR1 repeat units, and that this results in the production of less EBNA-LP [33]. This explains the apparent increase in EBNA-LP size and quantity in LPrev^i^, and may contribute to the reduced protein level in YKO, but does not explain the substantially increased EBNA-LP production by E2KO.

Resting adult B cells were infected with the recombinant viruses at equal titres to assess the abilities of viruses to transform B cells into LCLs. Three days post infection, E2KO-infected B cells were indistinguishable from uninfected cells, whereas all other viruses induced enlargement and aggregation of the cells (S6 Fig). After that time, LPKO^i^ showed very poor replication efficiency and usually failed to establish LCLs, whereas YKO consistently established LCLs. In the case where an LPKO^i^ LCL was established on feeder cells and maintained the LPKO^i^ genome (identified by episome rescue), EBNA-LP originating from an alternative EBV strain was detected (S7 Fig), suggesting that LPKO^i^ could be complemented by the virus strain endogenous to the B cell donor, but could not establish an LCL alone. Analysis of cell proliferation in the first week post infection confirmed a previous report that proliferation of EBV-infected cells begins over three days post infection [36], and showed that LPKO^i^ was very inefficient at driving cellular proliferation, while YKO was better (S8 Fig). However, LPrev^i^ was also poor at driving proliferation, being similar to YKO rather than being like WT^HB9^ and the other revertants (S8 Fig).

Since two independent pairs of LPKO^i^ and LPrev^i^ showed the same phenotype – LPKO^i^ was substantially more impaired than YKO (which itself produced so little truncated EBNA-LP protein, that it may be functionally a knockout) and LPrev^i^ exhibited defective transformation of B cells – we suggest that at least one of the intronic mutations in these viruses (ie the designed point mutation in the short intron, and/or the unintended minor variants in the BWRF1 ORF [33]) causes this defect. In order to assess the function of EBNA-LP without these confounding intronic IR1 mutations, a second method – Gibson assembly [37] – was used to produce a new recombinants of IR1, assembling repeat units that matched the B95-8 consensus between the flanking sequences from the B95-8 BAC (S9A Fig). One repeat array was assembled to produce a new wild-type IR1, and another containing the EBNA-LP mutation shown in Fig 1A. Thus the whole IR1 sequence (other than the defined EBNA-LP mutation) in these IR1 constructs matches the published B95-8 sequence (NC_007605). These repeat arrays were recombined into the IR1-knock-out to make two independent LPKO^w^ BACs (where ‘w’ indicates **w**ild-type IR1 backbone) and a repaired wild-type EBV with no IR1 heterogeneity (WT^w^) (Fig 1F). These were validated by pulsed field gel electrophoresis (S9B Fig) and used to generate virus-producing cell lines. Thus these new viruses represent a clean EBNA-LP knockout (LPKO^w^), and a wild-type virus (WT^w^) with a fully intact set of EBNA-LP exons.

### LCLs can be established using LPKO^w^ and its wild-type counterpart

**Fig 2:**
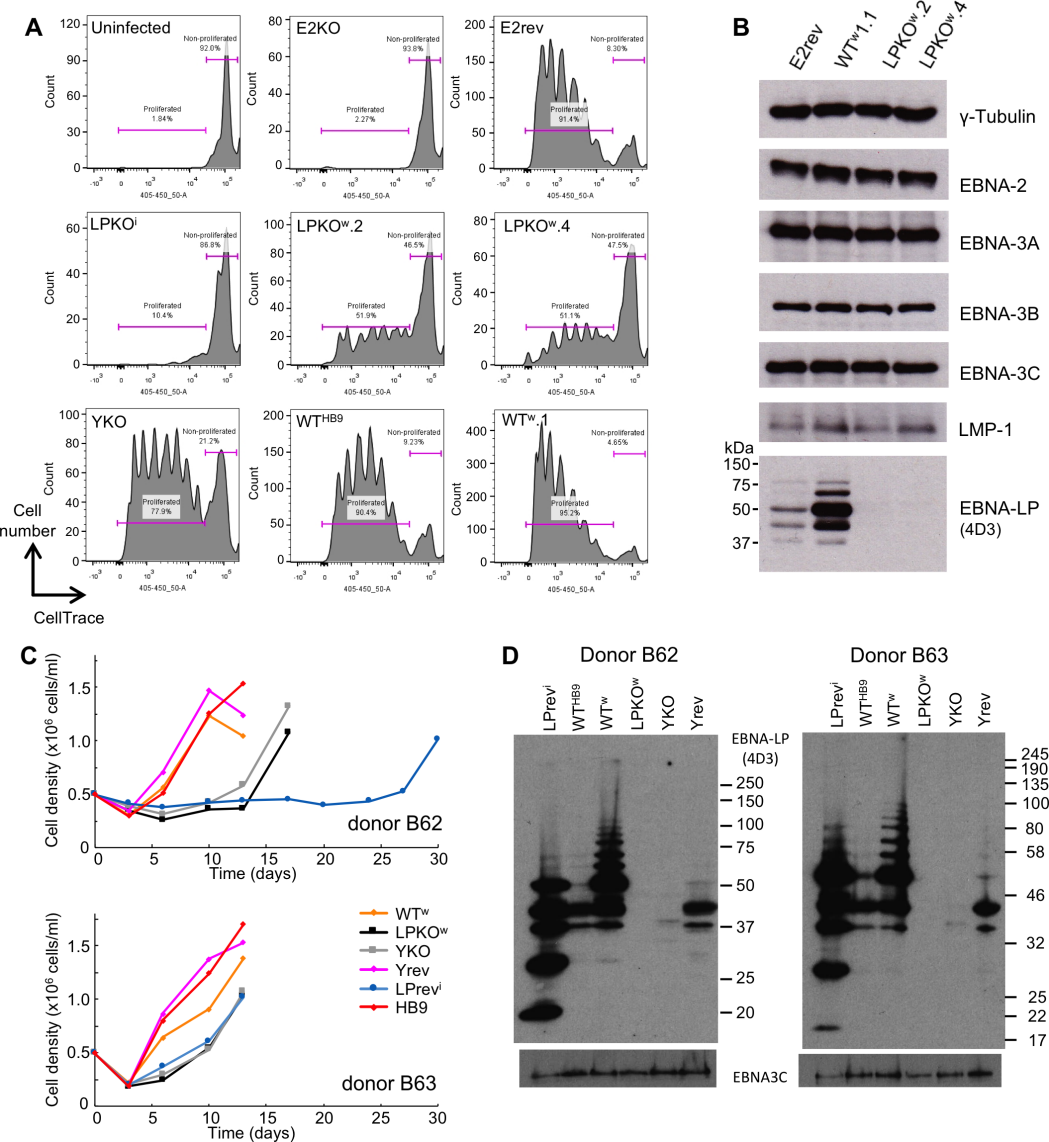
An EBNA-LP knockout EBV, LPKO^w^ is transformation competent. **A.** Cell proliferation of primary B cells 8 days post-infection, assessed by dilution of cell trace violet. The shown populations are gated for live single cells and CD20 positivity. **B.** Western blotting of viral proteins in LCLs established with LPKO^w^ and WT^w^ viruses. **C**. Cell counts to quantify outgrowth of 5x10^5^ adult B cells from two donors infected with various recombinant viruses (see key). Cells were disrupted and counted twice weekly. Cell count data are in S1 Data. **D**. Western blot showing EBNA-LP expression in LCLs established from the infections counted in C, with EBNA3C used as a loading control.

LPKO^w^ and WT^w^ were used to infect CD19-purified adult B cells alongside E2KO and YKO. Their proliferation after infection was assessed by dilution of the cell trace violet stain applied to the B cells prior to infection. As previously reported [36] EBV-infected cells did not divide until after day 3 post infection (S10 Fig). At 8 days post infection LPKO^w^ is superior to LPKO^i^ in driving infected B cells to undergo proliferation, approaching the level seen for YKO, suggesting that many of the important functions lost in LPKO^w^ are also missing in the YKO. WT^w^ matches (and perhaps exceeds) the transforming capability of the parental wild-type BAC and revertants (Fig 2A). LPKO^w^ and WT^w^ were both able to establish LCLs. Latency protein levels were largely similar between the LCLs, with LPKO^w^ LCLs clearly lacking EBNA-LP (Fig 2B), and WT^w^ showing an elevated level of EBNA-LP relative to the parental wild-type (WT^HB9^), due to the absence of a W1 mutation in a single IR1 repeat unit (Fig 2B and [33]).

LCLs could be reliably established from YKO, LPKO^w^ and LPrev^i^ (with weekly media changes that did not disturb cell clusters): robust expansion of B cell cultures infected by these mutant viruses was usually obvious from around 2 weeks after infection. To quantify this, 5x10^5^ adult B cells from peripheral blood of two donors were infected at an MOI of 1 (based on Raji green unit titre), and twice weekly the clumping cells were dispersed and cells counted (Fig 2C). While donors differed in how rapidly LCLs were established, EBNA-LP-deficient B cells grew out more slowly than the wild-type viruses. There was little or no difference between LPKO^w^ and YKO outgrowth, nor between WT^w^ and WT^HB9^ or Yrev (Fig 2C), even though WT^w^ expressed considerably more EBNA-LP (Fig 2D). LPrev^i^ outgrowth was more delayed than in previous experiments, and proliferated poorly when at low density, suggesting that its outgrowth was negatively affected by the repeated disruption of cells for counting. Western blotting confirmed the lack of EBNA-LP in LPKO^w^, the very low level of truncated EBNA-LP produced by YKO and the wider range of EBNA-LP isoforms produced by LPrev^i^ (Fig 2D).

### EBNA-LP mutant EBVs are defective in transforming cord blood B lymphocytes

**Fig 3:**
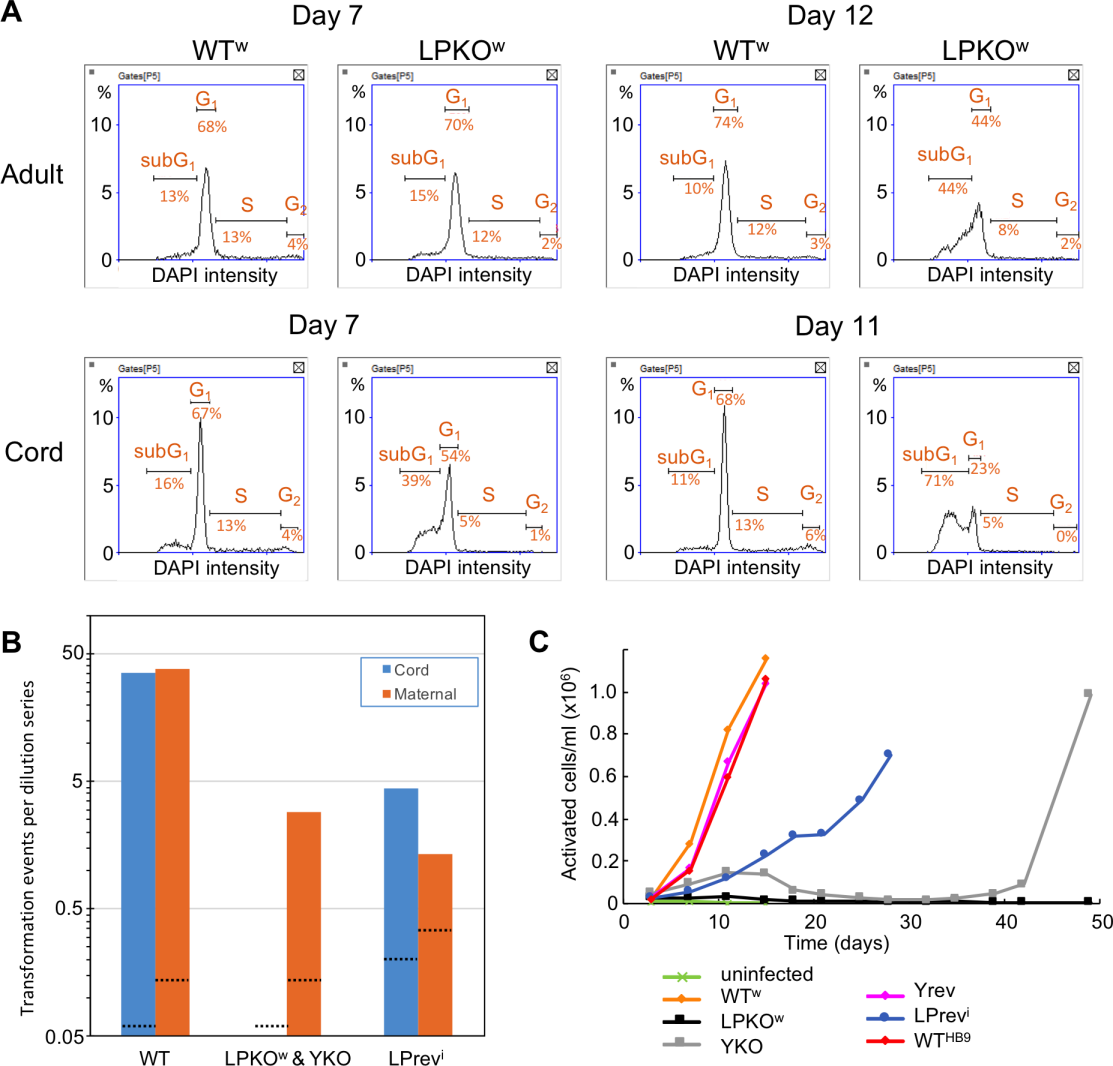
EBNA-LP mutants are defective at transforming B cells from cord blood. **A.** Cell cycle profiles of CD19+sorted adult and cord B cells infected with WT^w^ or LPKO^w^ viruses. Plots show the DNA quantity per cell (from DAPI staining). Quantities associated with different cell cycle phases are indicated. **B.** Transformation efficiencies for each infection were calculated from two-fold dilutions of infected cells (see Methods). These efficiencies were averaged for each virus group in each cell type across 3 (maternal–orange bar) or 5 (cord–blue bar) infections per virus. Black dotted lines indicate the sensitivity of the analysis for each virus–ie the calculated efficiency if only one well across all of the infections was positive. **C.** Outgrowth of LCLs from cord blood measured by counting the number of activated (ie large) cells. Cell count data are in S2 Data.

In addition to these infections of adult B cells, we also infected mononuclear cells from umbilical cord blood to try to establish LCLs. However, we were reproducibly unable to establish cord blood LCLs with the LPKO^w^ virus, regardless of whether CD19-purified B cells or total PBMCs were used, or if outgrowth was supported with irradiated MRC5 feeder cells. WT^HB9^, WT^w^, and importantly, LPrev^i^ – the severity of its transformation defect being similar to LPKO^w^ – were all consistently able to establish LCLs from cord blood. To investigate what was happening to the infected cells, we compared the cell cycle profile of B cells from umbilical cord blood with adult blood after LPKO^w^ and WT^w^ infection (Fig 3A). While more cell death is evident in LPKO^w^ than WT^w^-infected cells for both adult and cord blood (indicated by the subG1 population of cells) the difference is much more stark in cord cells, with both more dead cells and – by day 11 – far fewer cells in S or G2 phases of the cell cycle. By approximately 14 days post infection (precise timing varied with different donors), there were not enough live LPKO^w^-infected cord cells to analyse the cell cycle, and any remaining clumps of cells disintegrated and never recovered.

In order to quantify this transformation defect, we conducted a dilution cloning experiment comparing transformation of blood from the umbilical cord with blood taken at the same time from the baby’s mother. This was performed for three donor pairs, and LPKO^w^ and YKO viruses consistently failed to transform cord blood into LCLs, despite successfully transforming the maternal cells into LCLs (Fig 3B). In contrast, both the wild type viruses (WT^w^, Yrev and WT^HB9^) and LPrev^i^ showed no difference in transformation efficiency between cord and maternal lymphocytes. We then used cell counting to further characterize the outgrowth efficiency of the viruses in cord blood. Counting only cells that were larger than resting B cells (ie the activated, LCL-like cells) it was clear that while wild-type and LPrev^i^-infected cell numbers increased consistently after infection, YKO and LPKO^w^ infected cell numbers fell from around 10 days post infection (Fig 3C), consistent with the death of EBNA-LP-deficient cells at this time. In addition, WT^w^-infected cells expanded faster than the other wild-types (which make less EBNA-LP: Fig 2 and [33]). This faster expansion was also seen in the transformation assay. Since EBNA-LP-knockout EBV fails to transform cord blood cells, whereas the similarly deficient LPrev^i^ can, this failure does not represent a technical inability to establish cord LCLs with defective viruses in general. Therefore, we can conclude that EBV-transformed cord blood cells require EBNA-LP for their survival at a specific phase approximately 10–14 days post infection.

### EBNA-LP-deficient EBV is more defective in transforming adult naïve B cells than memory B cells

One key difference between adult B cells and cord-derived B cells is that the former are a heterogeneous mixture of different B cell subsets, whereas cord blood contains exclusively naïve B cells, as in utero the baby’s immune system has not yet encountered any pathogens. We therefore tested whether different adult B cell subsets differed in their susceptibility to transformation by LPKO^w^. B cells were sorted into subsets according to their CD27 and IgD status (S11 Fig): CD27-positive B cells are either class-switched (IgD^-^) or non-switched (IgD+) memory B cells; naïve B cells are CD27^-^/IgD^+^; and the double-negative population (CD27^-^/IgD^-^) is undefined, but is reported to have memory B cell-like properties [38]. All populations were isolated at 97–100% purity (S12 Fig). These populations (from six different donors) were infected with LPKO^w^ and WT^w^ EBV, and cultured to establish LCLs. Both WT^w^ and LPKO^w^ LCLs were established from the two memory B cell subsets of all donors tested, and (despite small cell numbers) the double negative population also established LPKO^w^ LCLs. Despite using the same MOIs and culture conditions, adult naïve B cells established WT^w^ LCLs more slowly than memory B cells. Nine attempts (from six donors) were made to transform naïve B cells with LPKO^w^ and only one (LC2) did so, and during outgrowth this culture exhibited a substantial loss of cells two weeks post infection.

**Fig 4:**
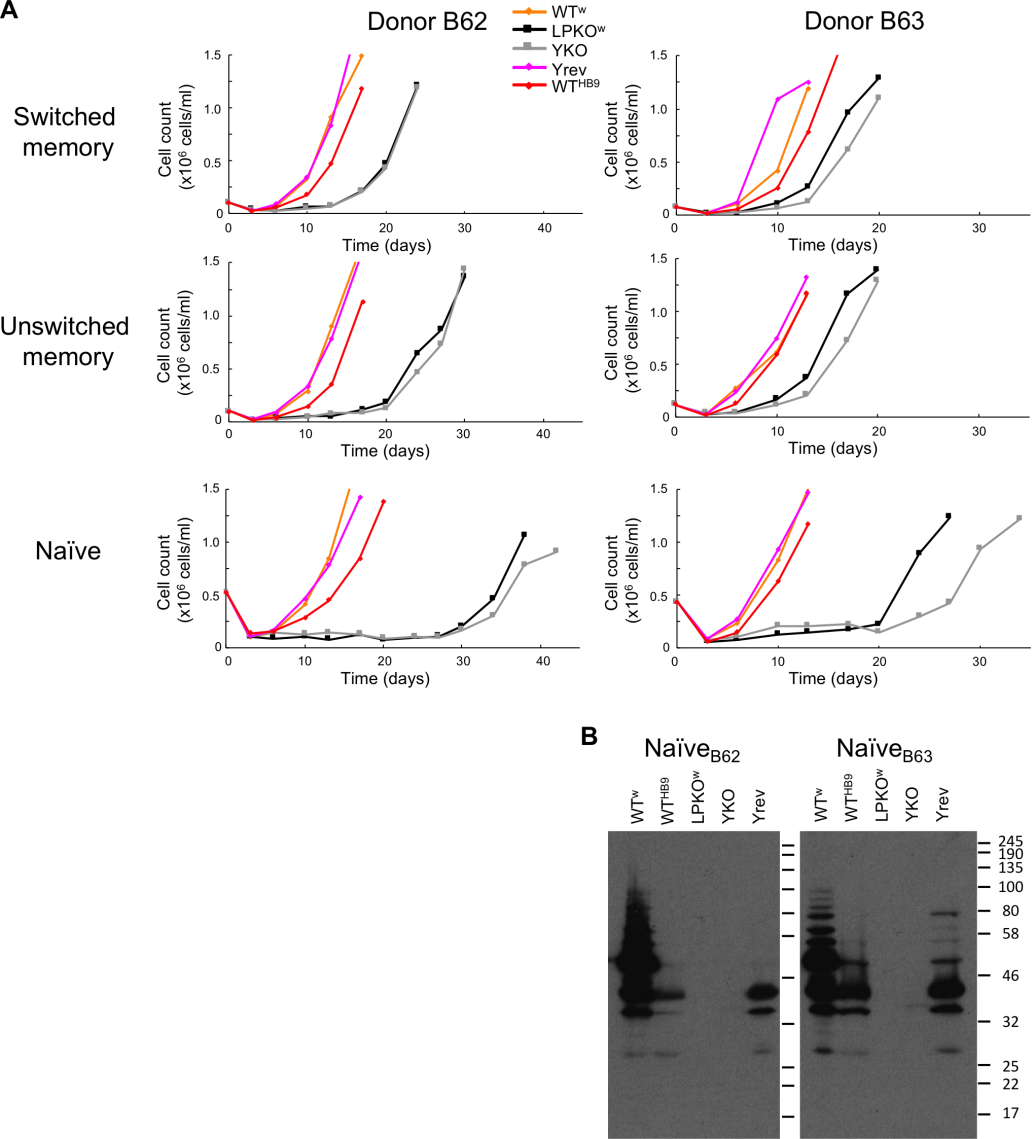
EBNA-LP mutants are less efficient at transforming adult naïve B cells than memory B cells. **A.** Cell densities counted during outgrowth of LCLs from different B cell subsets (indicated to the right) in two B cell donors (indicated top). The virus used is colour coded (key top centre), with EBNA-LP mutants indicated by monochrome lines, square markers, and wild-type viruses reddish shades, diamond markers. Outgrowth data are in S1 Data. **B.** Western blotting for EBNA-LP (antibody 4D3) confirms the expected EBNA-LP status of the LCLs.

To better quantify the outgrowth, two additional donors were sorted into subsets and infected with different virus mutants at an MOI of approximately 1 (although cell numbers varied according to the yield of cells of each population–S1 Data) and counted twice weekly (Fig 4A). For these two donors, LPKO^w^ and YKO were able to establish LCLs from the sorted naïve B cells, although they took considerably longer to grow out than from memory B cells, and exhibited a fall in the number of infected cells in weeks 2–3 before they grew out (S1 Data). We confirmed that these cell lines were not expressing EBNA-LP from a complementing virus (Fig 4B).

### EBNA-LP deficient LCLs always have a memory-like cell surface phenotype

**Fig 5:**
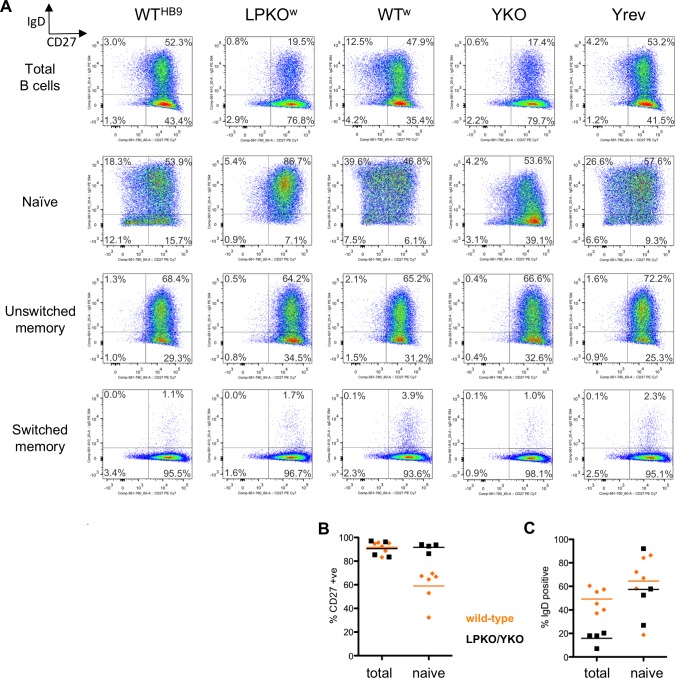
EBNA-LP-null LCLs only establish with a memory B cell-like phenotype. **A.** Flow cytometry plots show the CD27 (x-axis) and IgD status (y-axis) of LCLs established with different viruses (top labels) in different B cell subsets (labels left) from donor B63. Numbers in the plots show the percentage of cells in each quadrant. Naïve B cell-derived LCLs were all assayed on the same day, 39 days post infection. **B & C.** Graphs showing percentage of (**B**) CD27+ve or (**C**) IgD+ve cells from LCLs (based on data from Fig 5A and S13A Fig) produced from either naïve B cells or total B cells. Data points are shown as either EBNA-LP deficient (black square) or wild-type (orange diamond). Horizontal lines show means for each group.

It has been reported that the IgD status of an LCL matches that of the initially infected cell population [39]. We therefore measured the IgD and CD27 status of LCLs from the outgrowth experiments of total B cells and the different B cell subsets (Figs 5A and S13). The majority of the LCL cells established from mixed adult B cells were CD27-positive, reflecting the greater speed of transformation of memory B cells observed in Fig 4A. Wild-type LCLs established from naïve and unswitched memory cell subsets–expected to express IgD–were typically only two thirds IgD-positive suggesting that this mark is only modestly preserved in LCLs. In contrast, over 90% of cells from memory B cell-derived LCLs were CD27-positive.

The profiles of switched and unswitched memory B cell-derived LCLs were not different between EBNA-LP mutant and wild-type viruses, retaining the cell surface phenotype of the parental subset. In contrast all of the LPKO^w^ and YKO LCLs derived from naïve B cells had memory B cell profiles, having a consistently higher CD27 status than wild-type naïve LCLs (Fig 5B), although all LCLs have a much higher rate of CD27+ve cells than would be predicted by their starting profile. Similarly, YKO and LPKO^w^ LCLs from total B cells have a fewer IgD-positive cells than wild-type LCLs (Fig 5C), suggesting a preferential transformation of memory B cells by these mutants. Overall, the low outgrowth efficiency of naïve B cells infected with EBNA-LP mutants, and the memory-skewed profiles of EBNA-LP-deficient LCLs suggest that EBNA-LP is important for the survival of naïve B cells, a phenotype most robustly observable in cord blood LCLs.

### EBNA-LP facilitates the transcription of viral but not host genes

**Fig 6:**
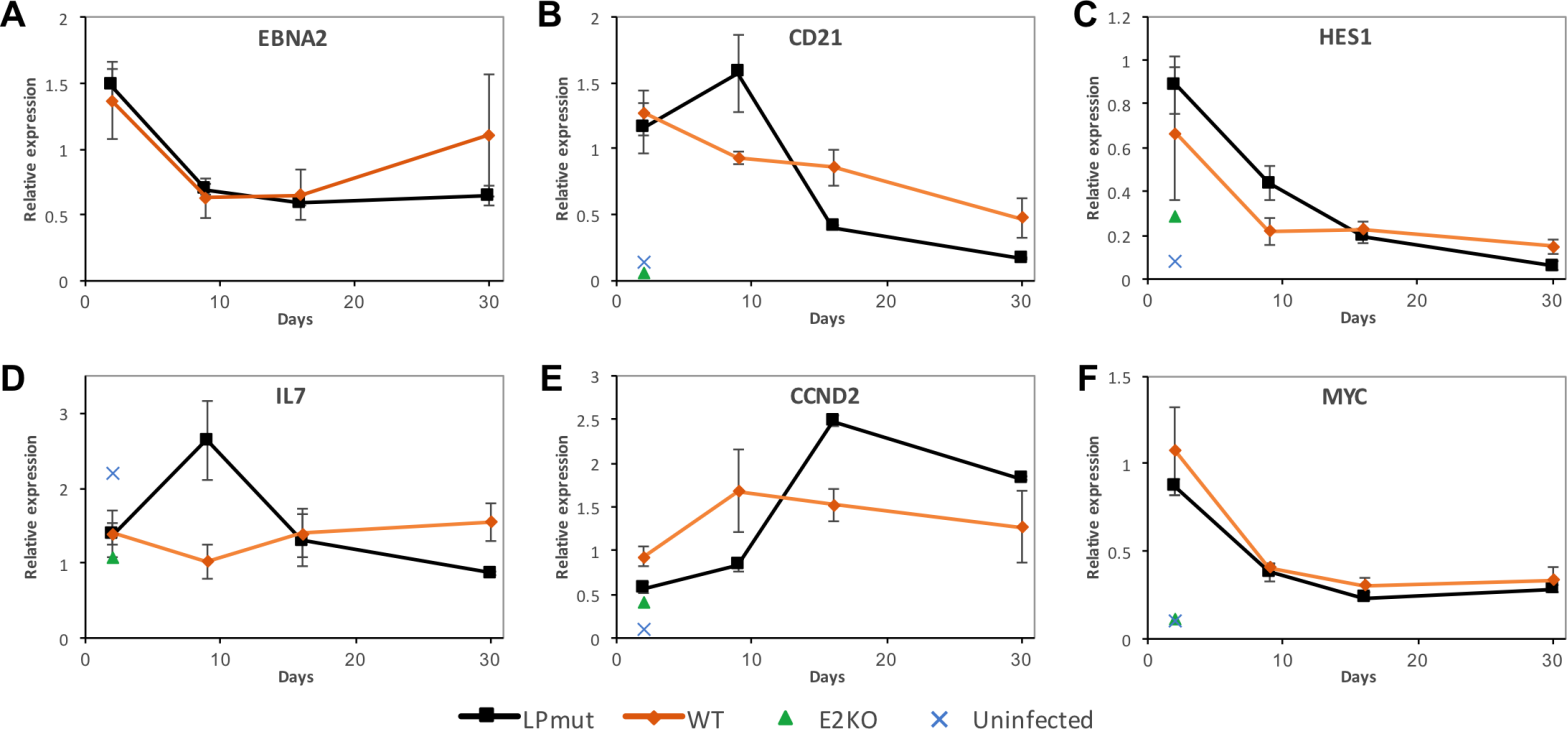
Activation of EBNA2-regulated host genes is not enhanced by EBNA-LP during EBV infection. Transcript levels (measured by qPCR) were normalised against endogenous control genes RPLP0 and ALAS1, and then expressed relative to the WT^HB9^ infection on day 2. Graphs A-F show levels of transcripts for the genes named within them as a time course after infection of resting B cells, grouped as either ‘wild-type’ (comprising WT^HB9^, WT^w^, Yrev, E2rev and LPrev^i^) or EBNA-LP mutants (LPKO^i^, LPKO^w^ and YKO) and compared with E2KO-infected and uninfected cells on day 2 – see key bottom. Error bars show ±1 standard deviation of the gene level for each group. Note that EBNA2 transcript level in E2KO infection was >10, so is omitted from that plot. Later time points have fewer EBNA-LP mutant samples as some knockouts did not survive. Complete data are in S3 Data.

It has been widely reported that cotransfection of EBNA-LP is able to enhance the transcription of viral and host genes induced by EBNA2 [7,23–27]. We therefore used qPCR to analyse host and viral transcript levels in two independent time courses across the first 30 days after infection of CD19 isolated mixed adult B cells at an MOI of 2. Comparing the EBNA-LP mutant viruses (LPKO^i^, LPKO^w^ and YKO) with the wild-type viruses (WT^HB9^, WT^w^, E2rev, Yrev and LPrev^i^) highlighted a number of differences between the groups that were consistent between the two time courses (S3 Data). *EBNA2* transcript levels were very similar across all infections (Fig 6A), except for E2KO, which had a 10-fold higher level transcript in both the EBNA2 and Wp assays (S3 Data). Since *EBNA2* levels were transcriptionally constant between EBNA-LP mutant and wild-type viruses (and by immunofluorescence EBNA2 protein levels were also no different–Fig 3C), we assessed the levels of host genes that are regulated by EBNA2. Two days post infection, known EBNA2-induced genes *CD21* and *HES1* [27,40] were not induced by E2KO, but were induced normally by the EBNA-LP mutants (Fig 6B and 6C), suggesting that EBNA-LP does not enhance the transcription of these genes by EBNA2 in the context of EBV infection. By day 9, transcription levels were higher in the EBNA-LP mutants than the wild-types – the opposite of what would be predicted from the transfection-based studies – for *CD21* and *HES1*, and also for *IL7* (Fig 6D), which is not induced by EBNA2 upon EBV infection, but does bind EBNA2 at its promoter [14], suggesting that in this context, EBNA-LP may restrain EBNA2’s transactivation ability. The cyclin D2 gene (*CCND2*), was the first EBNA2 target reported to be enhanced by EBNA-LP [7], but unlike the previous examples is indirectly activated by EBNA2 [41]. Its transcript levels may be higher in wild-type infections than EBNA-LP mutants (Fig 6E), but this effect is inconsistent between replicates (S2 Data). Any apparent effect of EBNA-LP on *CCND2* could alternatively be explained by the differences in proliferation induced by EBNA-LP mutants and wild-type EBVs, which could independently affect cyclin transcription. *MYC* activation by EBNA2 (which occurs both directly and indirectly [41]) is clearly unaffected by the absence of EBNA-LP (Fig 6F).

**Fig 7:**
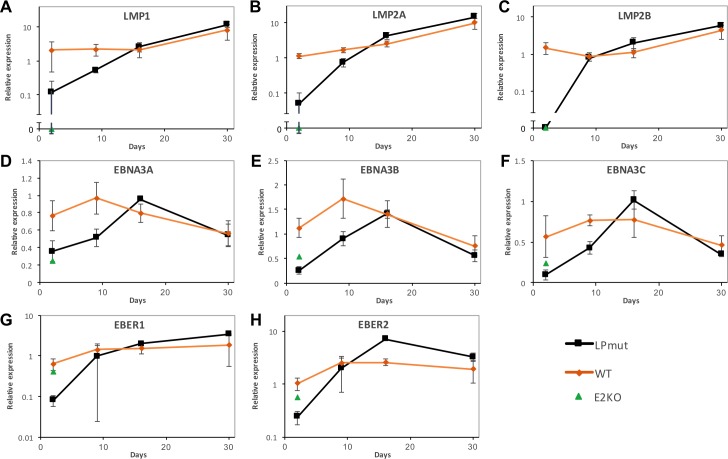
Viral latency genes are widely repressed in the absence of EBNA-LP. Graphs A-H show transcript levels of the viral gene named in each plot as a time course after infection of resting B cells, grouped as either ‘wild-type’ (WT^HB9^, WT^w^, Yrev, E2rev and LPrev^i^) or EBNA-LP mutants (LPKO^i^, LPKO^w^ and YKO)–see key bottom right. These are compared with E2KO-infected on day 2 only, as E2KO cells did not yield sufficient RNA at later time points. Transcript levels (measured by qPCR) are expressed relative to the level for the WT^HB9^ infection on day 2. Error bars show ±1 standard deviation of the transcript level for each group. Broken axes (in A-C) are used to allow zero values to be visualised on an otherwise logarithmic axis. EBNA3 transcript levels (D-F) are shown relative to the EBNA2 transcript level, since they share promoters, and E2KO Wp activity was very high. Later time points have fewer EBNA-LP mutant samples as some knockouts did not survive. Complete data are in S3 Data.

Having failed to support the idea that EBNA-LP enhances the transactivation potential of EBNA2 on host genes, we then investigated the expression of virus genes in these time courses. In keeping with the EBNA2 enhancement hypothesis, transcription of the LMP genes (*LMP1*, *LMP2A* and *LMP2B*) was delayed in EBNA-LP mutant EBV infections, reaching wild-type expression levels in the second week post infection (Fig 7A–7C). However, EBNA3 transcripts were also lower in abundance from EBNA-LP mutant EBVs (Fig 7D–7F). Since *EBNA3s* and *EBNA2* (which was unaffected by EBNA-LP) are transcribed from the same Wp and Cp promoters (see Fig 1B), we measured promoter usage, and observed an inconsistent tendency to have slightly more Wp and less Cp transcription in EBNA-LP mutants (S14 Fig and S3 Data). Most surprising was the failure of EBNA-LP mutants to promptly induce transcription of the EBER genes, which are not EBNA2 dependent (Fig 7G and 7H). Overall, we found that mutation of EBNA-LP led to a delayed transcription of all viral latency genes (other than *EBNA2*), regardless of whether they were EBNA2-dependent, but did not reduce the induction of host genes by EBNA2.

### EBNA-LP facilitates EBNA2 recruitment to viral but not host promoters

**Fig 8:**
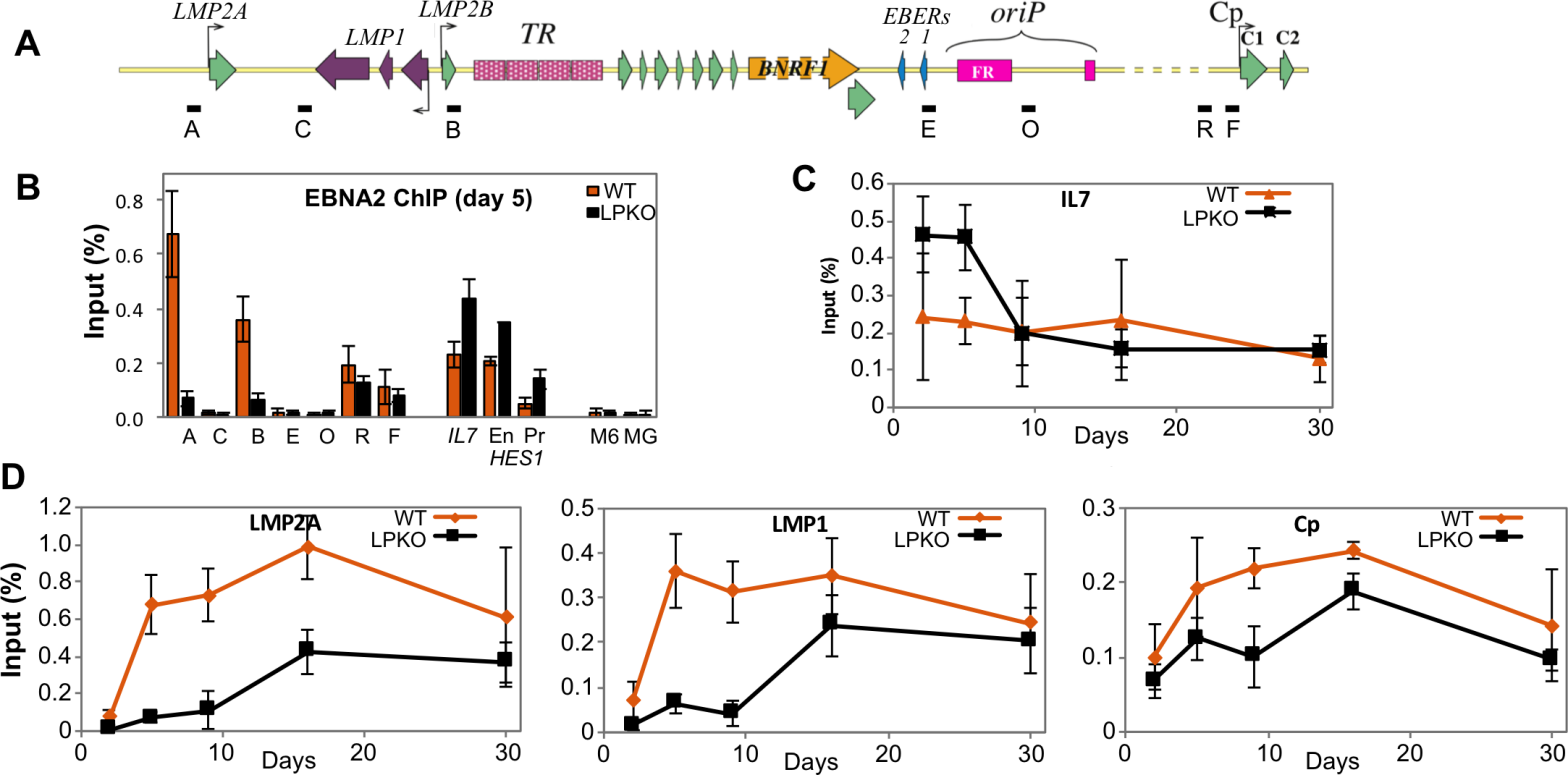
Binding of EBNA2 to viral and host loci is influenced by EBNA-LP. ChIP analyses of EBNA2 at promoters regulated by EBNA2 in LPKO^w^- and WT^w^-infected cells. **A.** Schematic representation of the EBV genome region containing latency promoters, showing positions of qPCR assays used for the ChIP. Dashed regions of the schematic are shown shorter than in reality. **B.** ChIP for EBNA2 in adult B cells five days post infection with WT^w^ (orange) and LPKO^w^ (black). Single letters are EBV locations per Fig 8A. EBNA2 binding sites in the host IL7 and the enhancer (En) and promoter (Pr) regions of HES1 genes are shown, with myoglobin (MG) and MCM6 (M6) genes as negative controls. **C.** Time course after LPKO^w^ and WT^w^ infection for EBNA2 binding to IL7 as a representative host gene locus. **D.** Time courses for viral promoters for LMP2A (assay A), LMP1/2B (assay B) and the RBPJK-binding site in Cp (assay R). All error bars in B-D show ± 1 standard deviation from the mean. Complete data sets are in S4 Data.

EBNA-LP has reportedly been detected by chromatin immunoprecipitation (ChIP) at various genomic loci, often in the presence of EBNA2 [42]. We have attempted to perform EBNA-LP ChIP, but have been unable to detect any difference in EBNA-LP ChIP-qPCR signal at either of the *LMP* promoters between wild-type and EBNA-LP knockout viruses in LCLs or during primary infections (S4 Data). Since EBNA2 has been repeatedly shown to bind to and regulate these genes, the binding of EBNA2 to both known binding sites and negative control sites (Fig 8A) was assessed across three 30 day infection time courses. The EBNA2 binding signal was low on day 2, so differences in EBNA2 binding were only sometimes detectable (S4 Data). By day 5 there is very little recruitment of EBNA2 to known transcription factor binding sites at both LMP promoters on the LPKO^w^ genome compared to WT^w^ (Fig 8B). EBNA2 recruitment to Cp was modestly reduced in LPKO^w^ infections, but still showed a considerable binding signal. In contrast, recruitment of EBNA2 to host genes *IL7* and *HES1* was more efficient after the LPKO^w^ infection, consistently showing elevated EBNA2 binding on days 2 and 5, but not at later time points (Fig 8C). The reduced binding of EBNA2 to the LPKO^w^ promoters was seen consistently for the first 2 weeks after infection (Fig 8D). The reduced detection of viral DNA in LPKO^w^ ChIPs is not due to there being less viral DNA in the LPKO^w^ cells, as the ChIP measures recovery as a percentage of input DNA, thereby controlling for variable input DNA levels. Thus these changes in EBNA2 binding are consistent with the lower expression of viral genes during the same period (Fig 7), and the elevated levels of host gene transcripts on day 9 (Fig 6B–6D).

### EBNA-LP facilitates host transcription factor recruitment to viral but not host loci

**Fig 9:**
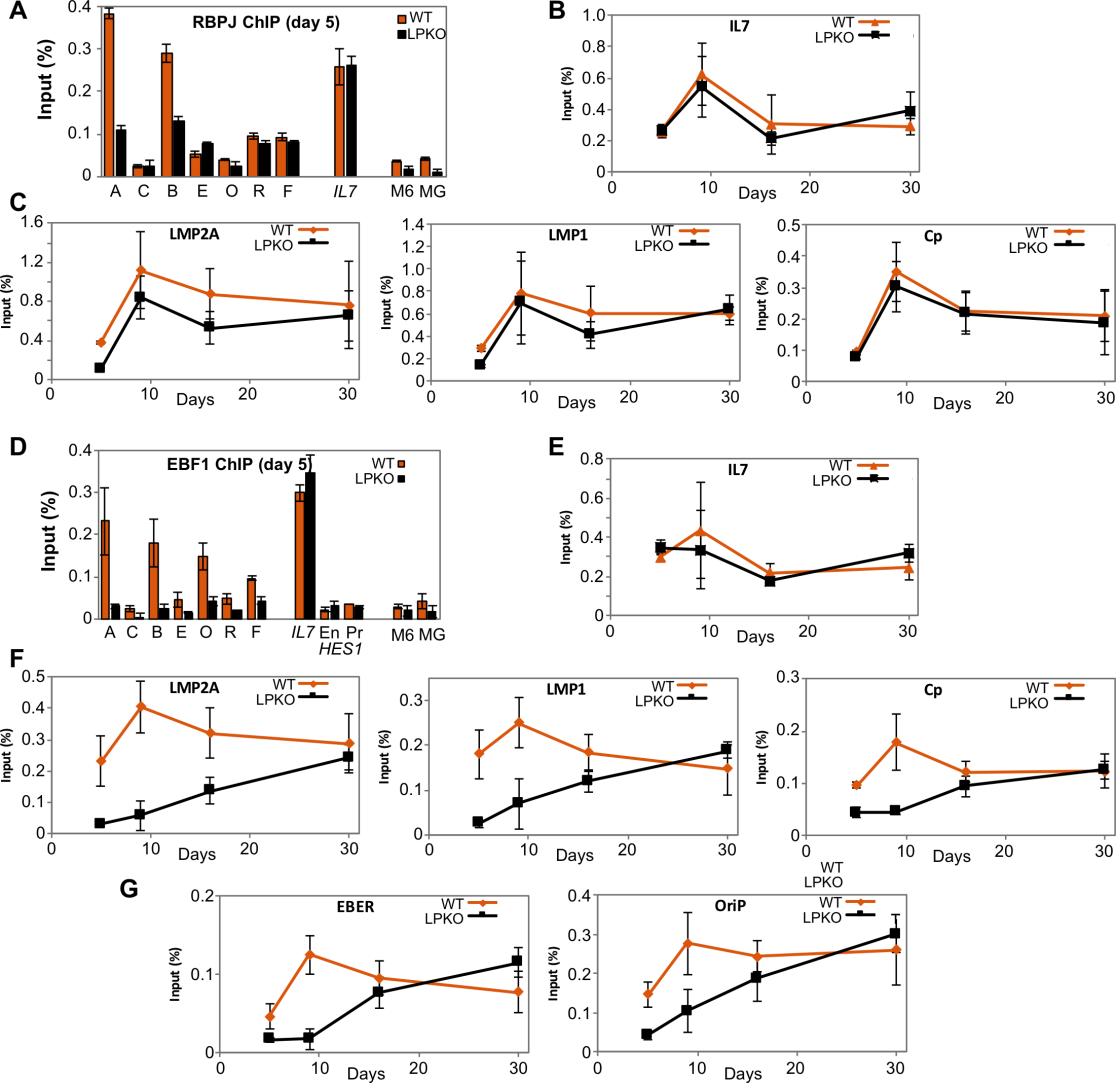
EBNA-LP enhances binding of host transcription factors RBPJ and EBF1 to viral but not host promoters. ChIP analyses of RBPJ (**A-C**) and EBF1 (**D-G**) binding at viral and host promoters after infection of adult B cells with LPKO^w^ (orange) or WT^w^ (black). ChIP for **A.** RBPJ and **D** EBF1 five days post infection at viral loci (single letters per Fig 8A) and genomic EBNA2/EBF-binding loci in the IL7 promoter and the HES1 enhancer (En) and promoter (Pr) regions, with myoglobin (MG) and MCM6 (M6) genes used as negative controls. **B and E.** Time course after LPKO^w^ and WT^w^ infection for RBPJ (**B**) and EBF1 (**E**) binding to IL7 as a representative host gene locus. **C and F.** Time courses for the binding of RBPJ (**C**) and EBF1 (**F**) to the EBNA2-binding regions of viral promoters for LMP2A (assay A), LMP1 (assay B) and the RBPJK-binding site in Cp (assay R). **G.** Time course for EBF1 binding to EBNA2-independent EBF1 binding sites in the EBER (assay E) and oriP (assay O) regions of the EBV genome. All error bars show ± 1 standard deviation from the mean. Full data sets are in S4 Data.

EBNA2 does not bind directly to DNA, but rather it is tethered to DNA by host proteins, in particular the transcription factor RBPJ (also called RBP-Jκ and CBF1). We therefore also assessed RBPJ binding to the viral and host loci. RBPJ binding was slightly (but consistently) lower in LPKO^w^ at day 5 post infection at the LMP promoters but identical at Cp and host locus IL7 (Fig 9A). No differences were observed on host genes at any time point (Fig 9B), or from 9 days on for virus genes (Fig 9C), suggesting that the apparent delay in RBPJ recruitment to the LPKO^w^ genome is slighter than EBNA2. However, it is notable that the peak of RBPJ recruitment to the viral genome (9 days) is later than the peak of EBNA2 recruitment (5 days) at all loci tested (Fig 9C compared to Fig 8D). This is consistent with a previous report that shows EBNA2 arriving at loci ahead of RBPJ [14].

Recent genome-wide analyses have shown that EBNA2 and RBPJ are often located with early B cell factor (EBF1) on the genome [42], and that the three proteins can bind together to chromatin [14]. In addition, EBF1 binds to two RBPJ-independent sites on the EBV genome, one near to the EBERs and the other near oriP. On day 5 post infection, EBF1 showed lower occupancy of all of its sites on the LPKO^w^ genome (Fig 9D), while the virus did not alter EBF1 binding to host genome loci at any time (Fig 9E). In wild-type infections, EBF1 binding reached maximal levels at all EBNA2-bound viral sites between 5 and 9 days post infection (Fig 9F), similar to EBNA2. Recruitment of EBF1 both to EBNA2-dependent *LMP* and *Cp* promoters (Fig 9F) and to the EBNA2-independent *EBER*/oriP loci was delayed in LPKO^w^ infection. It took at least two weeks to approach wild-type levels at all of the viral locations tested, the same dynamics as EBNA2 recruitment (Fig 8D).

Overall these gene regulatory observations show a widespread failure of the LPKO^w^ virus to support the activation of viral genes, and the recruitment of both viral and host transcription factors to the EBV genome, including to EBNA2-independent genes. In contrast, EBNA2-bound host genes exhibited a transient elevation of EBNA2 recruitment that was matched by a transient transcriptional activation. Together this suggests that EBNA-LP is required to facilitate the recruitment of transcriptional activators to the EBV genome, particularly between *LMP2A* and oriP, but is not required for the activation of host genes by EBNA2.

## Discussion

Many recent advances in the understanding of EBV biology have been underpinned by the ability to analyse gene functions in the context of EBV infection by modifying the virus genome. The genetic analysis of EBNA-LP function represents a greater technical challenge than other EBV genes, as EBNA-LP transcription can initiate within any one of the IR1 repeat units. We devised a strategy using Gibson Assembly [37] to seamlessly generate an array of 6 IR1 repeat units that can generate any combination of IR1 repeat units in any order. This could be used to assess whether the roles of the first, last or internal repeats may have different functions, such as testing the observation that the first Wp in the genome may be more important than the others [43].

Previous approaches to genetically assessing EBNA-LP function have been restricted to either truncation of the protein by removal of the C terminal Y exons [28,29] or deletion of increasing numbers of IR1 repeats, which affects Wp numbers and the sisRNAs as well as EBNA-LP [30]. Our attempt to produce a similar Y exon knockout (YKO) virus was likely impaired by the stop codon we recently discovered in one of the W1 exons of B95-8 IR1 [33]. The truncated EBNA-LP made by YKO is barely detectable, so it is not clear whether the LPKO-like phenotype of YKO is because the truncated EBNA-LP is defective, or because it is made at such a low level. It is also notable that the original WT^HB9^ BAC makes a much lower amount of EBNA-LP than the repaired WT^w^ virus (Fig 2, Fig 4 and [33]), which corresponds to a slightly reduced outgrowth rate, most noticeably in cord B cells.

The LPKO^i^ and LPrev^i^ viruses–made using type IIS restriction enzyme-based strategy–have demonstrated that apparently innocuous sequence changes in non-coding regions of IR1 can reduce transformation. These viruses contained 4 differences from the parental IR1. Three of these–which lie within the theoretical BWRF1 ORF–are found in a single IR1 repeat unit of B95-8 [33], while the fourth was introduced into the small intron between exons W1 and W2. Both of these regions are reported to give rise to stable intronic sequence RNAs (sisRNAs) [31]. The shorter of these (sisRNA1), which derives from the W1-W2 intron, is more robustly characterized, being detectable by Northern blot, qPCR and RNA-seq. However, it has recently emerged that the splicing of IR1 is more complicated than previously realised: transcripts for the anti-apoptotic Bcl2 homologue BHRF1 are spliced between W1 exons (skipping exon W2) during the lytic cycle. Thus, it remains possible that the LPKO^i^ and LPrev^i^ intronic mutation(s) may have disrupted non-EBNA splicing in IR1. However, we can conclude that the impact of the intronic mutation is EBNA-LP-independent, as LPKO^i^ is more defective than LPKO^w^, and LPrev^i^ is more defective than WT^w^. Further study is required to establish which intronic mutation is important, and what its mechanism may be.

### EBNA-LP is important for the transformation of naïve B cells

Our observations (Figs 3–5) suggest that EBNA-LP is far more important for the extended survival and proliferation of naïve B cells than memory B cells. This phenotype is clearest in cord blood, which invariably fail to survive more than two weeks after LPKO infection. For adult cells it is less clear. We have used CD27-negative, IgD-positive as a definition for naïve B cells in adult blood. The EBNA-LP-deficient LCLs established from this naïve population were all CD27+, and indeed many of the naïve B cell-derived wild-type LCLs contained substantial CD27+ populations. This may simply reflect a technical failure of the sorting process to remove sufficient memory B cells (although purity was over 98%) combined with a strong selective growth advantage for memory B cells after transformation. Alternatively, it is possible that in vitro EBV drives adult (but not cord) B cells from a naïve to a more memory-like phenotype. Whatever the characteristic of cord blood B cells that makes EBNA-LP essential, this characteristic is shared by most but perhaps not all adult CD27-/IgD+ cells. It may also be that CD27/IgD status does not accurately reflect a cord-like naïve phenotype. Indeed, CD27 is a somewhat flawed marker of B cell memory, since 5–25% of cord blood B cells are typically CD27-positive [44,45] despite the neonatal immune system never having met antigen, while CD27-/IgD- cells are reportedly a memory B cell subset [38].

Despite these uncertainties over a robust definition of naïve B cells, it is clear that EBNA-LP is essential for the survival of a naïve or cord-like B cell subset after transformation, but not for the survival of memory B cells. Since LPKO-infected umbilical cord B cells died two weeks after infection of both mixed lymphocyte and CD19-isolated B cells, the difference must be intrinsic to the B cell subsets rather than a bystander cell type. Another example of an EBV mutant exhibiting different transformation phenotypes in different B cell subsets, is shown by a BZLF1-knockout EBV being better able to transform germinal center B cells than memory or naïve B cells [46]. Nevertheless, the difference between naïve and memory cells is still surprising, as (at the transcriptome level) they are much more similar to each other than they are to germinal center cells [47].

Biologically, some differences have been reported that separate the behaviour of memory and naïve B cells. We noted a slower outgrowth of LCLs from naïve than from memory B cells, although this was not seen in a previous study [39]. Interestingly, adult naïve B cells enter cell cycle later than memory cells after CD40L stimulation [48], and produce fewer cells from such cultures [49]. In cord cells the defect is more profound, with CD40 agonism barely inducing any activation markers, whereas equivalent naïve B cell subsets from adults did respond [50]. Additionally, IgM crosslinking in cord cells failed to induce ERK1 phosphorylation, in contrast with adult cells [50], while BCR crosslinking on adult cells induced a larger response in memory than naïve cells [51]. These observations might be relevant, since the signals from antibody crosslinking and CD40 activation are also invoked by the LMP proteins [52]. LPKO viruses show delayed *LMP* gene activation, but the inference that the deregulation of LMPs in LPKO may be responsible for the death of naïve B cells is countered by the observation that this death mainly manifests around 10–14 days post infection, by which time LMP transcripts in LPKO infections are approaching wild-type levels (Fig 7).

Other phenotypic differences between naïve and memory cells may also contribute. For instance, IL2 stimulation enhanced the production of memory cells by CD40L, but not naïve cells [48], while 95% of cord B cells are negative for the IL2 receptor [45,50]. Naïve B cells also have a much lower level of the anti-apoptotic BCL2 family members MCL-1 and BCL-XL (but not BCL2 or Bim) than memory cells [53,54], which may contribute to the apoptotic phenotype of the LPKO^w^ naïve cells.

Together these reports suggest that naïve and memory B cells are phenotypically different, both in their response to pro-proliferative signaling and their resistance to apoptosis. What is less clear is how EBNA-LP overcomes these differences in naïve cells. EBNA-LP can interact with a complex of the tumor suppressors MDM2, p53 and the cyclin-dependent kinase inhibitor p14^ARF^ [55], which could influence both proliferation and apoptosis responses. It can also interact with the mitochondrial protein HAX1 [56], which can be either pro- or anti-apoptotic [57]. Alternatively, metabolic stress has been reported to be an important limitation to B cell transformation, and has been linked to an elevated EBNA-LP:EBNA3 ratio [58]. Furthermore, both EBNA-LP and EBNA3A have been shown to bind the prolyl-hydroxylase proteins that influence HIF1a stability, with the suggestion that this alters the metabolic state of the infected cells [59]. However, further study is required to understand the biology underlying the difference in transformation of naïve and memory cells, and to understand whether these differences are important for the in vivo biology and pathogenesis of EBV.

### EBNA-LP facilitates viral transcription, but does not widely enhance the transactivation of genes by EBNA2

The function of EBNA-LP has long been linked to EBNA2 because of their co-expression immediately after infection, and from a series of co-transfection experiments that appeared to show an enhancement of EBNA2’s transactivation function in the presence of EBNA-LP [7,23,24,26,27,40,60]. These studies demonstrated an ability to enhance transcription from reporter constructs [24,60], from host genes—most notably *HES1* and *CCND2* (Cyclin D2) [7,40]—and from EBV promoters repressed in the latency I transcriptional profile, including *LMP1* [23,27], *Cp* [60] and *LMP2A* [40]. Some studies have failed to replicate the regulation of some of these genes [27], but activation of the *LMP1/LMP2B* bidirectional promoter has been observed consistently. By taking a genetic approach to controlling the presence of EBNA-LP, and by analyzing gene expression in the context of viral infection rather than transfecting isolated EBV proteins, our approach differs considerably from previous studies. In doing so, we have made substantially different observations.

First we have not observed any conclusive examples of EBNA2-regulated host genes being enhanced by EBNA-LP. Outside transfection-based reporter assays, the only evidence of a direct relationship between EBNA2 and EBNA-LP comes from the observation that EBNA-LP lacking its acid-rich C-terminus (but not full-length EBNA-LP) can bind to EBNA2 [61], and that ChIP-seq experiments imply overlap between EBNA2 and EBNA-LP binding sites on the genome [42]. Our data are unclear as to whether (as previously reported [7]) EBNA-LP enhances the activation of *CCND2* (indirectly regulated by EBNA2 [41]), as the modest reduction in its transcription early after infection of EBNA-LP deficient cells could also be due to their reduced proliferation. In contrast, the transient increase in EBNA2 binding to *CD21*, *IL7* and *HES1* in EBNA-LP-deficient infections (Fig 8B and 8C) followed by a transient surge in their transcript levels (Fig 6B–6D), could be explained by a direct relationship between EBNA2 and EBNA-LP, but one where EBNA-LP restrains or modulates the transactivation of those genes, rather than enhances it. It is also possible that the increased levels of EBNA2 at the genes may be a consequence of the wider deregulation of the virus genome, either by modestly increasing the EBNA2 protein levels in the cell, or freeing up EBNA2 that would normally bind to the virus, leaving it available to bind to the host genome. Either way, these data suggest that whatever the relationship between EBNA2 and EBNA-LP, it is not simply one of enhancer and co-activator.

In contrast to host genes, transcription of both EBNA2-dependent (*LMP*s) and EBNA2-independent (*EBER*) genes is profoundly delayed in the absence of EBNA-LP, and there is also a widespread delay in the recruitment of transcription factors–both viral (EBNA2) and cellular (EBF1, and a lesser extent, RBPJ)–to the LPKO^w^ genome. EBNA transcription is also affected, and while *EBNA2* transcripts were not affected by the loss of EBNA-LP, the reduced levels of the *EBNA3* transcripts downstream suggest that the processing of the transcripts is different in the LPKO infection, since–like EBNA2 –they are also initiated at the Cp and Wp promoters. A similar elevated ratio of upstream to downstream EBNAs (as a ratio of EBNA-LP to EBNA3C protein levels) was observed in cells that have only proliferated 1–3 times after EBV infection [36], and in cells that arrest after an initial period of proliferation [58]. This could result from either an increase in polyadenylation after EBNA2, a change in splice site usage, or reduced elongation of transcripts. Indeed, there is evidence that the elongation complex pTEFb is important for transcriptional elongation from Cp, but is predicted to be less important for Wp [62], leading to speculation that elongation of Wp transcripts is less efficient, which would lead to lower yields of downstream EBNAs. Our data only weakly suggest a greater Wp and lesser Cp usage. Either delayed EBNA2 recruitment to Cp or reduced EBNA1 production (which might be similar to the EBNA3s, as they are similarly spliced) and binding to oriP could explain this difference.

A more profound effect was seen on the EBV latency genes between LMP2A and oriP (see schematic in Fig 8A). Activation of this whole genome region was severely delayed in EBNA-LP mutant infections, and this correlated with the delayed recruitment of EBF1, EBNA2 and – albeit less dramatically – RBPJK to the viral genome. The failure to induce transcription of the *EBER*s demonstrates that EBNA-LP is not simply working through EBNA2, or indeed through RNA polymerase II, as the *EBER*s are transcribed by RNA polymerase III, albeit regulated by transcription factors more typically associated with RNA polymerase II [63]. The region of latency genes from the *LMP2A* promoter to oriP represents a coordinately regulated genomic locus. It is flanked by CTCF binding sites [64], and these loop together to form a transcriptional unit. Disruption of the CTCF binding site near the *LMP2A* promoter can disrupt this loop, consequently reducing *LMP* gene transcription and increasing repressive histone and DNA methylation in LCLs [65]. The simplest interpretation is that EBNA-LP is important for the proper establishment of this transcriptional unit. There is a profound delay in the recruitment of transcription factors, but by 4 weeks post infection, the LPKO LCLs have reached normal levels of *LMP* and *EBER* transcripts, so there does not appear to be a defect in the maintenance of the locus once it is established in LPKO cells. EBF1 and RBPJ have been described as a pioneer factors: transcription factors that are able to access chromatinised DNA and establish new enhancer regions [14], so it is surprising that they would require EBNA-LP it to efficiently access the incoming EBV genome.

Notably, this region also includes the terminal repeats, and the virus – linear in the virion – needs to recircularize before LMP2 can be transcribed, and perhaps before this whole region is properly regulated. In addition the terminal repeats contain a binding site for PAX5, which is directed to the viral genome by EBER2 [66]. Two of the factors reported to bind to the EBER2/PAX5 complex (NONO and SPFQ) have also been found to bind to EBNA-LP in a tandem affinity mass spectrometry experiment [67], although both frequently exhibit non-specific interactions according to the CRAPome repository [68]. Thus it is possible that EBNA-LP is important for the efficient recircularisation of the terminal repeats, or for assembling the EBER2/PAX5 complex on them.

Rather than just the *LMP*/oriP locus, EBNA-LP could be relieving the repression of the viral genome as a whole. EBNA-LP binds to Sp100, a component of PML nuclear bodies (or ND10). In so doing, EBNA-LP transiently disperses Sp100 from ND10 [22], and subsequently localises to ND10 in LCLs [20]. ND10 are known inhibitors of herpesvirus early gene expression and lytic replication [21]. EBNA-LP – along with the EBV tegument protein BNRF1, which disrupts the ND10 component DAXX – is able to complement an ICP0-null herpes simplex virus, which otherwise is repressed by ND10 [69]. Thus it is reasonable to suggest that EBNA-LP is important for preventing the suppression of latency genes of EBV by ND10 via its interaction with Sp100, although if this is the case, it is unclear how EBNA2 transcription has evaded this effect.

Sp100 has also been implicated in the ability of EBNA-LP to enhance EBNA2-dependent gene transcription in transfection assays, so these phenomena may have a common origin. However, EBNA2 coactivation has also been attributed to EBNA-LP’s ability to bind to HDACs 4 and 5 [60], or NCOR [40]. EBNA-LP binding to NCOR and HDACs are both reported to sequester these repressive proteins away from EBNA2-inducible genes, thereby improving transactivation [40,60]. Any (or a combination) of these remain reasonable hypotheses as to how EBNA-LP facilitates viral transcription after infection.

In summary, we have undertaken a genetic analysis of EBNA-LP function and shown that EBNA-LP is important for B cell transformation, and essential for the transformation of naïve B cells, and that the role of EBNA-LP is far more complex than the previously proposed cofactor for EBNA2, being particularly important for establishing the viral transcription program. We also suggest that future analyses of EBV mutants would be better performed in distinct B cell subsets, as it is clear that phenotypes can vary considerably according the differentiation state of the infected B cells, and perhaps also the age of the B cell donor. The observations and genetic manipulation strategies described herein also extend approaches to study EBNA-LP, the EBV-sisRNAs and the wider functions of IR1 in the future.

## Methods

### Generation of recombinant EBVs

In order to introduce mutations into IR1, we have devised a strategy for introducing a constructed IR1 repeat into EBV. This entails first deleting the virus’s endogenous IR1 (to prevent the constructed repeat from recombining with the original one) and then inserting the rebuilt repeat. To achieve this, we used RecA-mediated recombineering as previously described [70]. The viral IR1 was deleted by joining together homology regions from the unique (non-repetitive) sequences flanking IR1: The upstream region (NC_007605 positions 11413–12008), which contains exon C2, was cloned SfiI/PciI from the B95-8 BAC (clone WT^HB9^); the downstream region (position 35239–35869) was cloned XhoI/MluI. This region was introduced by recombineering in place of IR1. The same homology regions were used as flanks for newly assembled IR1 repeats containing EBNA-LP mutations.

We have used two distinct methods to generate the synthetic IR1. Both approaches generate an IR1 with 6.6 copies, which is a typical size for circulating EBV strains [32] and is the size of IR1 in the parental EBV-BAC clone, WT^HB9^. In both cases, the IR1 was assembled in a pBR322-based plasmid in DH5alpha bacteria grown at 30°C to reduce unwanted recombination.

The first approach used to assemble a modified IR1 adapted a strategy that used type IIs restriction endonucleases to assemble tandem repeats [71–73]. A BamW fragment was subcloned from the B95-8-BAC clone WT^HB9^ into a vector that contains binding sites for the type IIb restriction endonucleases BsmBI and BtgZI. These restriction sites were engineered to both cut at the site of the BamHI restriction site (S1B Fig). A DNA fragment (between the MfeI and AgeI restriction sites in BamW) was synthesized, containing a point mutation of the BsmBI restriction site in the intron between exons W1 and W2 (S1A Fig), and also containing mutations that introduced STOP codons and a PvuI restriction site (Fig 1C), for making the EBNA-LP knockout virus, LPKO^i^. A second synthesized fragment containing the BsmBI mutation but not the EBNA-LP mutation was also synthesized for producing the revertant virus, LPrev^i^. These fragments were cloned into the BamW repeat unit, and then both the LPKO^i^ and LPrev^i^ repeat units were assembled into an array using the method described in S1C Fig. The array was then incorporated into independent IR1 knockouts [WKO] according to the scheme shown in Fig 1F, generating two independent LPKO^i^ viruses, and their revertants. Unintentionally, the BamW fragment used to make these viruses contained three sequence differences from the B95-8 consensus that are present as minor variants in B95-8, found in one of its IR1 repeat units [33].

Subsequent recombinant viruses that contained changes in IR1 were made without the need to mutate the BsmBI restriction site in the W1-W2 intron, and using a BamW fragment that matched the B95-8 consensus sequence. A BamW repeat unit was cloned into a pBR322-based plasmid that contained BtgZI restriction sites that cut the BamHI sites flanking the repeat unit. This was then modified with oligonucleotide linkers on either (or both) sides of the BamW fragment, such that the BamW sequence was extended approximately 20bp from the BamHI restriction site (S9A Fig). To generate the wild-type IR1, the constructs were cut and assembled as shown in S9A Fig, and the assembly was cloned into pKovKan and recombined into the WKO.4 that had been used to produce LPKO^i^.2, thereby generating WT^w^.1 (see Fig 1F). To generate the new EBNA-LP knockout (LPKO^w^) the BsmBI point mutation in the synthesized LPKO region was reverted to the wild-type sequence by InFusion mutagenesis, and subcloned into the new wild-type BamW fragment. The IR1 synthetic array was then assembled in the same way as the wild-type array, and used to independently generate LPKO^w^.2 and LPKO^w^.4 viruses by recombineering into WKO.4. E2KO, E2rev, YKO and Yrev BACs were generated by RecA-mediated recombineering essentially as described elsewhere. The precise sequences of the E2KO and YKO deletions are shown in Fig 1. Revertants were made by reintroducing wild-type sequence into the knockouts by the same method (Fig 1F).

BACs were screened for integrity using EcoRI, AgeI, HindIII, NotI and BamHI restriction digests and run on a CHEF DRII chiller pulsed field gel electrophoresis system (Bio-Rad). We noted that the family of repeats (FR) region of oriP is smaller in WT^HB9^ than predicted by sequence. This reflects a previous observation that the family of repeats region (FR) of oriP is unstable, even in BACs, and that the FR in the p2089 BAC (of which WT^HB9^ is a subclone) is 300 bp smaller than the authentic sequence of B95-8 [74]. Therefore, in addition to restriction digests, all recombinant BACs were screened by PCR, using the KA2 and KA3 primers [75] with Q5 DNA polymerase (NEB) to ensure that the FR region was the same size in all recombinants.

### Generation of producer cell lines and virus

Recombinant EBV BAC DNA was purified from bacteria by alkaline lysis followed by cesium chloride density gradient centrifugation. DNA was assessed by pulsed field gel electrophoresis to ensure a predominance of intact supercoiled BAC DNA, as DNA integrity appears to influence the number and quality of producer cell lines. The BAC DNA was transfected into 293-SL cells (a culture of the HEK-293 cell line provided by Claire Shannon-Lowe; University of Birmingham) using a peptide 6 and lipofectin transfection reagent described previously [76]. Cells were selected with hygromycin and colonies isolated by ring cloning. Individual hygromycin-resistant colonies were screened for GFP expression, for their ability to produce virus. The integrity of episomes from the producer lines was assessed by recovery into bacteria [77] and analyzed by restriction digest and pulsed field gel electrophoresis. Cell lines were only used if at least 80% of recovered episomes were indistinguishable from the parental BAC.

To generate virus stocks, 293-EBV producer cell lines were seeded in 10 cm dishes and after 1–2 days these were transfected at approximately 25% confluency with equal quantities of BALF4 and BZLF1-expressing plasmids: 12 μg total DNA per 10 cm plate when transfecting with peptide6+lipofectin or 6 μg per plate using GeneJuice reagent (Merck-Millipore). Supernatant was harvested after 5 days and filtered through a 400 nm syringe filter. Virus titer was assayed by infecting 2x10^5^ Raji cells in 1.5 ml with 10-fold dilutions of virus. After two days, 0.5 ml media supplemented with 20 ng/ml TPA and 5 mM sodium butyrate was added to the Raji cells, and left overnight, to enhance GFP expression in the infected cells. Cell clumps were dispersed by pipetting and total number of green cells per well were counted under a fluorescence microscope. This gave a Raji green units (rgu) titer, which was typically in the range of 0.5-10x10^5^ rgu/ml in the cell culture supernatant.

### Cell culture, isolation and analysis of immune cells and virus infections

LCLs, BL31 cells (provided by Alan Rickinson, University of Birmingham), and 293-SL cells were grown in RPMI media supplemented with L-glutamine (Life Technologies) and 10% fetal calf serum. Sera were batch tested for the ability to establish 293-SL-EBV-BAC colonies after BAC transfection, and to support outgrowth of LCLs under limiting dilution. MRC5 foreskin fibroblasts (ATCC CCL-171), also grown in RPMI, were irradiated with 50 Gy and seeded as a confluent monolayer to support outgrowth in some experiments.

Adult primary lymphocytes were isolated mainly from buffy-coat residues provided by NHS Blood and Transplant. Cells concentrated from a 500 ml original blood volume were diluted to 200 ml with PBS. Lymphocytes were isolated by layering blood-derivative on ficoll followed by centrifugation. The isolated peripheral blood leukocytes (PBLs) were washed twice in RPMI/1%FCS. B cells were purified from PBLs by hybridizing to anti-CD19 microbeads (Miltenyi), using 0.5ml beads per 10^9^ PBLs, followed by positive selection (possel program) on an autoMACS separator (Miltenyi). Either purified B cells or PBLs were resuspended at 1-2x10^6^ cells/ml in RPMI/15% FCS. B cell purity was measured by FACS for CD20 positivity, and was typically over 90%.

For isolation of different adult B cell subsets, the CD19-sorted B cells were rested overnight in a cell culture incubator, and then stained with fluorescent antibodies (from Biolegend) against IgD (PE-CF594, clone IA6-2) and CD27 (PE-Cy7, clone M-T271). The cells were sorted using a BD FACSAria III (BD Biosciences) into naïve and memory populations based on IgD and CD27 status as indicated in S11 Fig, and purity was assessed by counting a few hundred of the isolated cells on the FACSAria III (S12 Fig). Cell populations were counted and resuspended in RPMI/15% FCS at 2x10^6^ cells/ml for infection. CD27 and IgD status of LCLs was assessed using the same antibodies and staining protocol, measured on an LSRFortessa flow cytometer and analysed using FlowJo software.

Isolated PBLs or B cells were infected within a few hours of isolation/purification, by adding virus at an MOI of 1–2 rgu/B cell, and shaking at 37°C for 3 hours, after which cells were centrifuged at 200g for 10 minutes and seeded at a density of 1-2x10^6^ cells/ml in RPMI supplemented with L-glutamine and 15% FCS (batch tested for LCL outgrowth–GE healthcare or Life Technologies) and either 50 ng/ml (for purified B cells) or 500 ng/ml (for mixed lymphocytes) of cyclosporin A. During outgrowth, approximately half of the media volume was replaced every 5–7 days (cyclosporin A was omitted after two weeks), harvesting up to half of the cells, depending on experiment.

### Transformation and outgrowth assays

Blood from the umbilical cord and maternal blood was drawn from healthy full-term pregnancies with written informed consent of the mother (an adult) prior to the onset of labour, overseen by the UK National Health Service Research Ethics Committee (approval REC 13/LP/1712). Mononuclear cells were isolated from paired 0.5–2 ml blood samples of maternal and cord blood by ficoll gradient centrifugation. Variations in the yields of mononuclear cells meant that different infections were performed with different numbers of cells: two of the three donors used equal cell numbers for maternal and cord blood infections (3.4x10^5^ and 1x10^5^ cells per infection). The third pair used 5x10^4^ maternal cells, and triplicate infections of 3x10^5^ cord cells for each virus. For most viruses (LPKO^w^.4; WT^HB9^; WT^w^; LPrev^i^; YKO.4 and Yrev.4) 10^5^ Raji infectious units were used for each dilution series. LPKO^w^.2 was used at 10^6^ rgu per dilution series, but this higher titer showed the same transformation efficiency as LPKO^w^.4.

Each infection (and an uninfected control well) was placed in a well of a 96 well plate, and then serially diluted 2-fold ten times in RPMI/15% FCS/Cyclosporine A (100ng/ml). Media was changed weekly and after 6 weeks the number (n) of wells containing LCLs was counted, and number of transforming events per infection calculated as 2^(n-1)^.

Quantitation by cell counting was undertaken by infecting B cells (typically 5x10^5 cells–see S1 Data and S2 Data for exceptions) at an MOI of 1 in falcon tubes for three hours, and centrifuging and resuspending the cells in in 1ml RPMI containing 15% FCS in a 48 well plate. Twice weekly, cells were dispersed by vigorous pipetting, and 50 ul was taken for counting. Once per week (immediately prior to a count), 400 μl of media was removed, and replaced with 500 μl of fresh media, to feed the cells and ensure that the volume of culture was maintained. For counting, 2.5 ul of solution 13 (Chemometec)–which contains Acridine Orange to mark all cells, and DAPI to stain dead cells–was added to the 50 μl of cells. Ten μl of cells were added to an NC-slide A8 and cells automatically counted by fluorescence on a nucleocounter NC3000 (Chemometec). Using Nucleoview NC-3000 software (Chemometec), counted events were gated for size to exclude debris smaller than resting cells, and counts plotted both for total cells, and for activated cells only (defined as those with a larger Acridine Orange area than resting B cells–S1 Data and S2 Data).

### RNA analyses and quantitative reverse transcript PCR

For the time courses after infection of primary B cells, the cells were supplemented with an equal volume of fresh media 24 hours prior to harvesting. Then, half of the culture was taken (typically 5x10^5^ to 2x10^6^ cells) and RNA was extracted using RNeasy mini columns (Qiagen). For all samples in a time course, the same quantity of RNA (~300ng) was reverse transcribed using either Superscript III First-Strand Synthesis SuperMix for qRT-PCR (Life Technologies) 3 μl cDNA was mixed with TaqMan gene expression mastermix (Life Technologies) applied to a custom TaqMan low density array (TLDA) card containing duplicate assays (S1 Table), which used ALAS1, RPLP0, GNB2L1 and 18S RNA as endogenous control genes. EBV TaqMan assays were designed by Applied Biosystems/Life Technologies using proprietary software, and validated using B95-8 cDNA. Sequence information is proprietary. The assay IDs in S1 Table can be used to obtain these assays. The EBNA2 TaqMan assay extends from exon Y2 to downstream of the exon Y3 splice donor. It therefore also detects EBNA2 transcripts in the E2KO virus, which retains these sequences. The EBNA3 TaqMan assays were designed spanning the exon junction between the U exon and the first exon of each EBNA3. LMP exon junctions detected by LMP assays are shown in S1 Table. Additional assays (primers in S2 Table) were conducted using Kapa qPCR SYBR kit (low ROX), and the IL7 TaqMan assay used Takyon low ROX Probe 2X MasterMix dTTP (Eurogentec) and normalised against ALAS1 and RPLP0. Quantitation of qPCR data was performed using the delta-delta-Ct method, using DataAssist Software v3.01 (Thermo Fisher Scientific). All quantitation is expressed relative to the level for WT^HB9^ on day 2 post infection. Bulk PCR of transcripts across IR1 was performed using Q5 polymerase (NEB) and Cp-forward or Wp-forward primers with U-reverse or Y2end-reverse primers (S2 Table).

### Chromatin immunoprecipitation (ChIP)

ChIP was carried out using the Chromatin Immunoprecipitation (ChIP) Assay Kit (Millipore) according to manufacturer’s instructions. Briefly, 2x10^6^ infected B cells were fixed for 10 minutes in 1% formaldehyde and neutralised with glycine. After two PBS washes, cells were lysed with SDS Lysis buffer on ice for 10 minutes and sonicated using the Diagenode UCD-200 Bioruptor for 15 minutes. Precleared chromatin, using 45μl protein A agarose beads was diluted with ChIP dilution buffer and incubated overnight with primary antibodies against EBNA 2 (Abcam ab90543), EBF1 (Millipore AB10523), RBPJk (Abcam ab25949) or an IgG control (Sigma). Protein A agarose beads collected the immune complexes, which were subsequently washed in low salt, high salt, lithium chloride and twice in TE buffers. The immune complexes were eluted from the beads using elution buffer and left overnight at 65 degrees. After proteinase K treatment for 2 hours at 50 degrees, DNA was then purified using the Qiagen QIAQuick gel extraction kit, and eluted in 120 μl water.

Chromatin was quantified by qPCR using the Kapa qPCR SYBR kit (low Rox) on a QuantStudio7 real time PCR machine (Applied Biosystems). Primers used for ChIP have been described previously [78] [79] [14] [80] [62], and are listed in S3 Table. Absolute quantity (relative to input) was calculated from standard curves generated from input DNA that was serially diluted 1:4, four times. 2 μl of ChIP sample was amplified in triplicate for each qPCR assay.

### Dye-dilution-based proliferation assay

Prior to infection, primary cells were resuspended at 10^6^ cells/ml in PBS containing 5 μM CellTrace Violet (Life Technologies) and incubated for 20 min at 37°C in dark. This was then diluted 5 times in complete B cell media and incubated for 5 min at room temperature in the dark. Cells were washed by centrifugation and resuspended in fresh pre-warmed complete B cell media for infection. We noted that CellTrace violet staining had a variable propensity to kill primary B cells, so individual tubes were tested for toxicity by staining PBLs and comparing B cell percentage with and without staining. Tubes exhibiting less than 50% loss of B cells were used in experiments. For assay, cells (a volume equivalent to 10^6^ cells in the initial infection) were harvested on ice and stained for CD20-PEVio770 (Life Technologies), and resuspended in PBS/1%BSA containing DRAQ7 live/dead cell stain (BioStatus). Cells were analysed on a FACS machine (BD LSR II or LSRFortessa) and cell proliferation visualised for live CD20^+^ singlet cells using FlowJo software.

### DNA fragmentation assay

Approximately 10^6^ infected B cells were resuspended in 50 μl PBS and added to 450 μl of ice cold 70% ethanol and stored until all samples had been harvested. After least 12 hours in this ethanol, cells were pelleted by centrifugation at 500g for 5 minutes, resuspended in 1 ml PBS, incubated for 1 minute, pelleted, and resuspended in 100 μl PBS containing 1% triton X-100 and 1μg/ml DAPI. 30 μl of cell suspension was transferred to a NC-Slide A2 and imaged in a nucleocounter NC-3000 (Chemometec)

### Western blotting and immunofluorescence

Western blotting was performed as described previously, using RIPA lysates and run and blotted onto nitrocellulose using the mini-Protein systems (Bio-Rad). Antibody clones used were: EBNA-LP (clones JF186 [81] or 4D3 [82];gift from Yasushi Kawaguchi]); EBNA2 (Clone PE2); EBNA3A (Ab16126, Abcam); EBNA3B (Rat monoclonal 6C9 [17]); EBNA3C (mouse monoclonal A10); LMP1 (monoclonal CS1-4, Dako). For immunofluorescence, cells were grown on a 12 chamber slide (Ibidi). Cells were gently washed with PBS and then fixed with 4% paraformaldehyde for 15 minutes. Cells were washed twice with PBS and covered with blocking buffer (PBS/10% FCS/100mM glycine/0.2% Triton X-100) for 30 minutes. Cells were stained with primary antibody in 50 μl blocking buffer for one hour, washed thrice in PBS and stained with fluorophore-conjugated secondary antibody (Cheshire Bioscience) for an hour. Chambers were washed three times with PBS and then the chamber removed, the slide briefly dipped in deionized water, and a coverslip mounted on the slide with Prolong Gold Antifade mount with DAPI (Life Technologies). Slides were imaged on a Zeiss LSM5 Pascal confocal microscope: 63x objective, 4x digital zoom and shown as a projection of z-stacks of 1μm sections.

### Ethics statement

Adult blood cells were purchased from UK National Blood and Transplant as waste products of platelet isolation. As they are waste products from anonymous volunteer donors, no ethics approval is required. Umbilical cord blood (and the maternal blood) were obtained with written informed consent of the mother (an adult) prior to the onset of labour, under the MatImms study, approved by the UK National Health Service Research Ethics Committee (approval REC 13/LP/1712). Anonymized blood samples surplus to the requirements of the MatImms study were used in this project, distributed by the Imperial College Healthcare NHS Trust Tissue Bank (REC 12/WA/0196) and approved by the tissue bank’s Tissue Management Committee (project R15029). Other investigators may also have received these same samples.

## Supporting information

S1 Fig Generation of viruses containing recombinant IR1 using type IIS restriction enzymes. **A.** Intronic mutation introduced to remove a BsmBI restriction site in the intron to allow this cloning method to be used. **B.** Method for the construction of LPKO^i^ and LPrev^i^ viruses. Type IIS restriction enzyme sites used to assemble repeat arrays were designed to cut at the same site as BamHI in a pBR322-based plasmid. The BamHI sub-fragment (BamW) was subcloned into the BamHI site in the orientation indicated. The internal BsmBI restriction site that was mutated to allow this construction method is outlined by a green box. Other features of the IR1 repeat are indicated. **C.** Cloning strategy for the assembly of LPKO^i^ is shown. LPrev^i^ assembly followed an equivalent series of cloning steps. Grey boxes indicate BamW fragments, while white boxes indicate the SfiI/BamHI or BamHI/MluI regions at the edges of IR1 as shown in A above. Black box within BamW represents the mutation of EBNA-LP and the deleted BsmBI restriction site. Plasmid IDs are indicated. C and Y indicate the exons at the flanks of the targeting region.
(PDF)

S2 Fig Pulsed field gel analysis of recombinant EBVs. Analyses show the diagnostic digests for the construction of: **A.** LPKO^i^ and its revertand LPrev^i^; **B.** E2KO and E2rev; **C.** YKO and Yrev. The size standard marker (M) is a 1:1 mixture of BstEII-lambda and Lambda mono-cut marker (NEB). **A.** Recombinant LPKO^i^ and LPrev^i^ viruses are identical, including all containing 6.6 IR1 repeats, other than bands altered by the inserted PvuI restriction site or removal of BsmBI. Digestion at these sites results in conversion of the IR1 band (white arrow) into the 3kb IR1 repeat unit (green arrow) and the Cp and Y bands flanking the repeat (yellow arrows). **B.** Size changes in E2KO result from introduction of EcoRI and PvuI restriction sites. **C.** YKO mutation produces a 140bp reduction in band size that is too small to detect in these digests, and an introduced EcoRI restriction site that causes a more easily observed change (red arrows). All other bands are unchanged, demonstrating the integrity of the genome outside the intended mutations.
(PDF)

S3 Fig Recombinant EBV validation in BL31 cells. **A.** To test whether the splicing of EBNA transcripts had been affected by the changes inserted into the viruses, PCRs were conducted between the C1 and W0 exons (upstream) and the YH exon downstream to compare the transcripts produced by wild-type EBV and the LPKO^i^, LPrev^i^, and YKO EBVs. **B.** Western blotting of EBV protein levels in BL31 cells stably infected with the various recombinant viruses. A and B suffixes indicate independent BL31 cell lines produced from the same virus.
(PDF)

S4 Fig Western blot validation of EBNA2 knockouts in BL31 cells. Various western blots for EBV proteins in cell lines infected with EBNA2 knockouts and revertants. Each lane is identified by the virus recombinant, above the identifier of the 293 cell virus producer line, and bottom is the BL31 cell line ID. Each lane therefore represents an independent cell line. Note that BL31-E2KO-GK is a cell line generated using a different EBNA2-knockout EBV, produced by Gemma Kelly and Alan Rickinson [34].
(PDF)

S5 Fig Immunofluorescence analysis of EBNA2 and EBNA-LP expression after infection of primary B cells 48 hours post infection. Antibodies used to label proteins are shown as indicated. EBV-infected cells were reproducibly seen associated with pericellular foci that were labelled by the anti-mouse secondary antibody alone. These are indicated by purple arrows. Yellow arrows indicate an apparently nucleolar accumulation of the truncated EBNA-LP in YKO infections. The red single channel image in YKO has been brightened to improve visualisation of the faint EBNA-LP signal. Other channels use the same brightness across the experiment. Note the extremely intense staining of EBNA-LP in E2KO infected cells.
(PDF)

S6 Fig Transformation of B cells by recombinant viruses. Photographs of the accumulation of transformed cells after infection of CD19-purified B cells by various EBV strains, taken on days 2–20 post infection as indicated. Activated cells form clusters that then proliferate to differing extents.
(PDF)

S7 Fig Western Blot characterisation of LPKO^i^, LPrev^i^ and YKO-established LCLs. Western blots of proteins from LCLs grown out from recombinant EBV infections. The virus used for the outgrowth is indicated. Initial phase of the outgrowth of cells was either performed on irradiated MRC5 feeder cells (F) or without feeder cells (N). The epitope in EBNA-LP recognised by the JF186 antibody exists in B95-8 but is missing from most virus strains. Antibody 4D3 recognises all known EBNA-LP variants.
(PDF)

S8 Fig Induction of proliferation by recombinant viruses. Flow cytometry plots from live CD20-positive cells harvested either **A.** 3 days, **B.** 5 days or **C.** 7 days after infection of adult B cells stained with CellTrace Violet prior to infection. Degree of dilution of the violet signal is indicated on the x-axis, indicating number of cell divisions. Proliferation of infected cells was measured by dilution of CellTrace violet.
(PDF)

S9 Fig Generation of recombinant viruses containing an IR1 repeat produced by Gibson assembly. **A.** Schematic representation of the Gibson assembly strategy used to generate LPKO^w^ and WT^w^. Grey boxes represent the BamW fragment and white boxes the flanks of the repeat as described in S1 Fig. Red and orange arrows indicate the sequences either side of the BamHI restriction site within IR1. These arrows are the homology regions whose overlap drives the Gibson assembly of overlapping fragments as indicated in the lower part of the figure, which shows the assembly of wild-type BamW fragments into the IR1 used to generate WT^w^. To generate LPKO^w^, the mutated W exons were cloned into each of the five plasmids indicated, and the assembly performed in the same way. **B.** Pulsed field gel analysis of the recombinant WT^w^ and LPKO^w^ viruses compared to the parental WT^HB9^. The PvuI digest shows the presence of the knockout-specific mutation in EBNA-LP (yellow arrows), releasing multiple copies of the 3kb IR1 repeat unit (white arrow), as compared to the parental BAC (WT^HB9^) and WT^w^. The other digests show the overall integrity of the rest of the virus genome.
(PDF)

S10 Fig Proliferation of cell lines at various time points. Flow cytometry plots from live CD20-positive cells harvested either 4, 11 or 15 days after infection of adult B cells stained with CellTrace violet prior to infection. Degree of dilution of the violet signal is indicated on the x-axis, indicating number of cell divisions. Proliferation of infected cells was measured by dilution of CellTrace violet. Data for day 8 are found in Fig 3A.
(PDF)

S11 Fig Gating strategy used to define B cell subsets. Example flow cytometry plot of magnetically purified B cells (CD19+ve) stained with CD27 and IgD. Coloured boxes show the position of the gates used to separate the four subsets. IgD^-^/CD27^-^ B cells (Magenta) are not a functionally defined subset (or could be non-B cells), so have not been named.
(PDF)

S12 Fig FACS sorter and purity plots. Plots show the IgD (y-axis) and CD27 (x-axis) status for the pre and post-sort populations from six donors, with donor ID shown in the top of each red box. For each donor, the top two plots show the pre-sort population (total events left; smaller number of events right). The lower plots for each show the identity of each sorted population with the percentage purity shown as magenta numbers.
(PDF)

S13 Fig EBNA-LP-null LCLs only establish with a memory B cell-like phenotype. **A**. Flow cytometry plots show the CD27 (x-axis) and IgD status (y-axis) of LCLs established with different viruses (top labels) in different B cell subsets (labels left) from donor B62. Numbers in the plots show the percentage of cells in each quadrant, used to plot Fig 5B and 5C. Naïve B cell-derived LCLs were all assayed on the same day, 46 days post infection. **B.** Similar analysis of LCLs established from donors LC1 and LC2 (where memory-derived LCLs were from a mixture of switched and unswitched memory B cells).
(PDF)

S14 Fig QPCR analysis of Cp, Wp and total LMP2 transcription. Time course of transcript levels based on Taqman qPCR assays spanning: **A**. Exon W0 to W1 to indicate activity of the Wp promoters; **B**. Exon C1/2 to W1 to measure Cp promoter activity; and **C**. spanning exons 5 and 6 of LMP2 that measures LMP2 transcripts originating from LMP2A exon 1, LMP2B exon 1, and terminal repeats that should constitute all LMP2 transcripts.
(PDF)

S1 Table TLDA card Taqman qPCR assays. (XLSX)

S2 Table Primers used for PCR and qPCR. (XLSX)

S3 Table Primers used for ChIP-qPCR. (XLSX)

S1 Data Cell count data for LCL outgrowth from donors B62 and B63. The first tab (Expt set up) describes the virus stocks used, their titre, the cell numbers per well used for each B cell subset, and the amount of virus used, with resultant MOI (between 1 and 2) shown at the bottom of the table. Numbers 1–6 (code number column) are the labels found in the raw data identifiers. B cell subset is indicated in row 5: B = total B cells; N = Naïve; UM-unswitched memory; SM = Switched memory, defined according to IgD/CD27 status.Cell numbers per sample (row 17) for the three subsets represents 1/6 of the total yield of cells from the sorting. The raw data from the NC3000 is in the final tab of the workbook. Each count measured the total live cells (gated by acridine orange area to remove debris), and also separated the cells into two sizes—the size that matched all of the uninfected (ie smaller) cells—gate M2—and a gate for the larger cells, that mainly appeared only after EBV infection. The second tab contains two sets of data and plots: the left part of the tab is the total DAPI-ve cells (gate M2+M3: live small + live large); to the right is plotted only the larger live (DAPI-ve) cells (gate M3; live large cells only). Each set of data is plotted on linear and log scale graphs to help visualise different aspects of the data.
(XLSX)

S2 Data Cell count data for LCL outgrowth from cord blood B cell sample C282. The first tab (Expt set up) describes the virus stocks used, their titre, the cell numbers per well used for each B cell subset, and the amount of virus used, based on an MOI of 0.6, shown at the bottom of the table. Numbers 1–6 (code number column) are the labels found in the raw data identifiers. Cell numbers per sample (row 15) represents 1/7 of the total yield of cells from the magnetic sorting of B cells from 7 ml of cord blood. The raw data from the NC3000 is in the final tab of the workbook. Each count measured the total live cells (gated by acridine orange area to remove debris), and also separated the cells into two sizes—the size that matched all of the uninfected (ie smaller) cells—gate M2—and a gate for the larger cells, that appeared only after EBV infection. The second tab contains two sets of data and plots: the left part of the tab is the total DAPI-ve cells (gate M2+M3: live small + live large); to the right is plotted only the larger live (DAPI-ve) cells (gate M3; live large cells only). Each set of data is plotted on linear and log scale graphs to help visualise different aspects of the data.
(XLSX)

S3 Data Primary B cell infection qPCR data. The first tab contains the data from time course 2, which is shown in the figures in the main body of the paper. Time course 1 is shown in the second tab. Differences in outgrowth speed, between the two time courses makes it hard to meaningfully combine the data sets. Individual data measures are shown in the left set of tables. Averages and Standard deviation by groups are in the right hand tables. Data in the right hand table are shown normalised to endogenous control genes, and expressed relative to the expression level for WT^HB9^ on day 2. For some plots on log scales, 0 values have been changed to the lowest value on the plot (these plots are shown with broken axes in the paper figures). These changed data are shown as red text—original data are retained in the left hand tables.
(XLSX)

S4 Data Chromatin immunoprecipitation (ChIP) data. These tabs contain the individual ChIP data points and summaries used to produce Figs 8 and 9. The Expt Summary tab contains a list of all ChIPs performed for EBNA2, EBF1 and RBPJ. Each B cell infection number (column C) represents a distinct B cell donor. Time shows the time after infection that the cells were fixed and chromatin extracted. Column E shows which ChIPs were performed on each batch of extracted chromatin. The grid to the right shows which qPCRs were performed for each batch of extracted chromatin. The identities of the qPCRs in row 4 match the labels in Fig 8A. The next three tabs are summaries for each of the three ChIPs, which contain the data and plots for time courses used in the Figs 8C, 8D and 9B, 9C, 9E, 9F and 9G. The next tab—Day 5—is the data for Figs 8B, 9A and 9D, which shows all assays on day 5. The subsequent tabs are the results of each independent ChIP experiment, split into tabs by ChIP Antibody (EBNA2, RBPJ or EBF1) and time point (Day 2, 5, 9, 16, 30). A few data sets (numbers in red text in the various tabs) were excluded from the plotted data due to technical failings with that experiment or sample. These are included for full disclosure (and generally show the same trends).
(XLSX)
