# Correction: Epstein-Barr virus nuclear antigen EBNA-LP is essential for transforming naïve B cells, and facilitates recruitment of transcription factors to the viral genome

**DOI:** 10.1371/journal.ppat.1007403

**Published:** 2019-02-21

**Authors:** Agnieszka Szymula, Richard D. Palermo, Amr Bayoumy, Ian J. Groves, Mohammed Ba abdullah, Beth Holder, Robert E. White

In the original submission, the negative control in ChIP experiments was misidentified as detecting the myoglobin promoter: it in fact detects the myogenin promoter. As a result, there are textual errors in the captions for Fig 8, “Binding of EBNA2 to viral and host loci is influenced by EBNA-LP,” and Fig 9, “EBNA-LP enhances binding of host transcription factors RBPJ and EBF1 to viral but not host promoters.” There is also an ambiguity in the second table in the S3 Table file that has been clarified.

**Fig 8:**
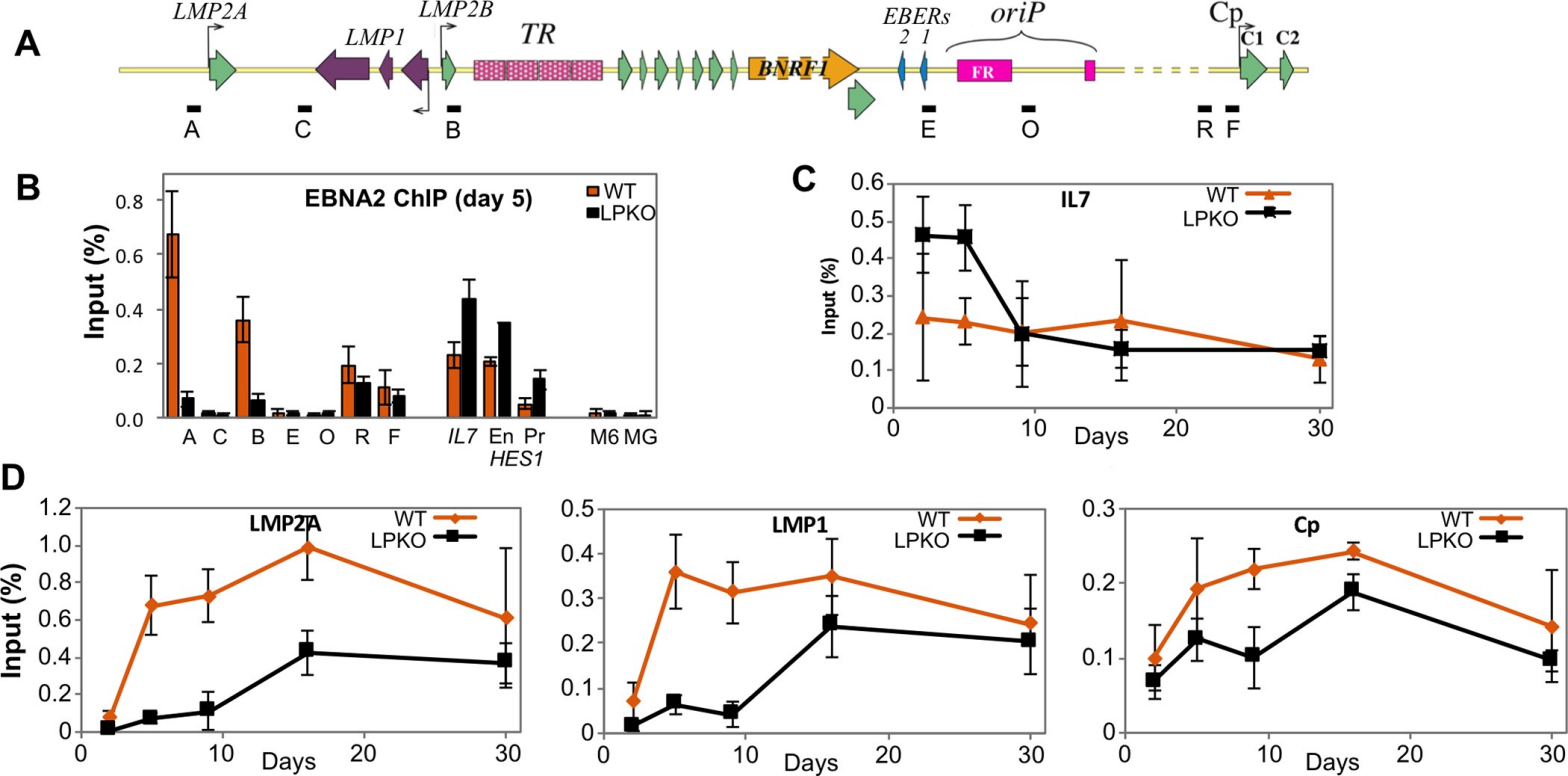
Binding of EBNA2 to viral and host loci is influenced by EBNA-LP. ChIP analyses of EBNA2 at promoters regulated by EBNA2 in LPKO^w^- and WT^w^-infected cells. **A.** Schematic representation of the EBV genome region containing latency promoters, showing positions of qPCR assays used for the ChIP. Dashed regions of the schematic are shown shorter than in reality. **B.** ChIP for EBNA2 in adult B cells five days post infection with WT^w^ (orange) and LPKO^w^ (black). Single letters are EBV locations per Fig 8A. EBNA2 binding sites in the host IL7 and the enhancer (En) and promoter (Pr) regions of HES1 genes are shown, with myogenin (MG) and MCM6 (M6) genes as negative controls. **C.** Time course after LPKO^w^ and WT^w^ infection for EBNA2 binding to IL7 as a representative host gene locus. **D.** Time courses for viral promoters for LMP2A (assay A), LMP1/2B (assay B) and the RBPJK-binding site in Cp (assay R). All error bars in B-D show ± 1 standard deviation from the mean. Complete data sets are in S4 Data.

**Fig 9:**
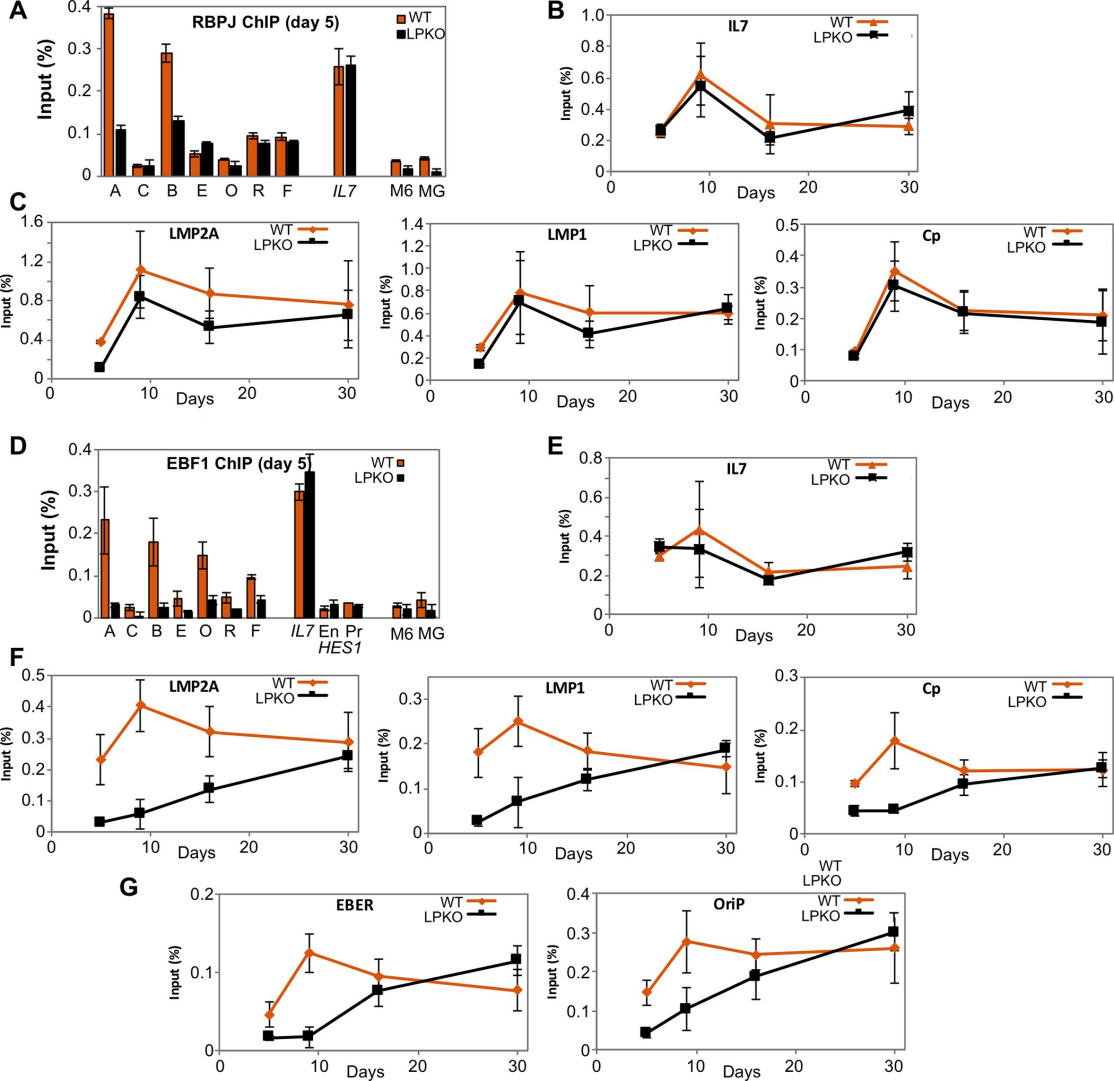
EBNA-LP enhances binding of host transcription factors RBPJ and EBF1 to viral but not host promoters. ChIP analyses of RBPJ (**A-C**) and EBF1 (**D-G**) binding at viral and host promoters after infection of adult B cells with LPKO^w^ (orange) or WT^w^ (black). ChIP for **A.** RBPJ and **D** EBF1 five days post infection at viral loci (single letters per Fig 8A) and genomic EBNA2/EBF-binding loci in the IL7 promoter and the HES1 enhancer (En) and promoter (Pr) regions, with myogenin (MG) and MCM6 (M6) genes used as negative controls. **B and E.** Time course after LPKO^w^ and WT^w^ infection for RBPJ (**B**) and EBF1 (**E**) binding to IL7 as a representative host gene locus. **C and F.** Time courses for the binding of RBPJ (**C**) and EBF1 (**F**) to the EBNA2-binding regions of viral promoters for LMP2A (assay A), LMP1 (assay B) and the RBPJK-binding site in Cp (assay R). **G.** Time course for EBF1 binding to EBNA2-independent EBF1 binding sites in the EBER (assay E) and oriP (assay O) regions of the EBV genome. All error bars show ± 1 standard deviation from the mean. Full data sets are in S4 Data.

Please view the correct S3 Table and the complete, correct captions here.

## Supporting information

S3 Table Primers used for ChIP-qPCR. (XLSX)
