# Supplementary material for: Epstein-Barr virus nuclear antigen EBNA-LP is essential for transforming naïve B cells, and facilitates recruitment of transcription factors to the viral genome
Source: PLoS Pathog. 2018 Feb 20;14(2):e1006890. doi: 10.1371/journal.ppat.1006890 (PMC5834210; doi:10.1371/journal.ppat.1006890)
Supplement: S2 Fig — Analyses show the diagnostic digests for the construction of: A. LPKOi and its revertand LPrevi; B. E2KO and E2rev; C. YKO and Yrev. The size standard marker (M) is a 1:1 mixture of BstEII-lambda and Lambda mono-cut marker (NEB). A. Recombinant LPKOi and LPrevi viruses are identical, including all containing 6.6 IR1 repeats, other than bands altered by the inserted PvuI restriction site or removal of BsmBI. Digestion at these sites results in conversion of the IR1 band (white arrow) into the 3kb IR1 repeat unit (green arrow) and the Cp and Y bands flanking the repeat (yellow arrows). B. Size changes in E2KO result from introduction of EcoRI and PvuI restriction sites. C. YKO mutation produces a 140bp reduction in band size that is too small to detect in these digests, and an introduced EcoRI restriction site that causes a more easily observed change (red arrows). All other bands are unchanged, demonstrating the integrity of the genome outside the intended mutations. (PDF) [file ppat.1006890.s002.pdf]

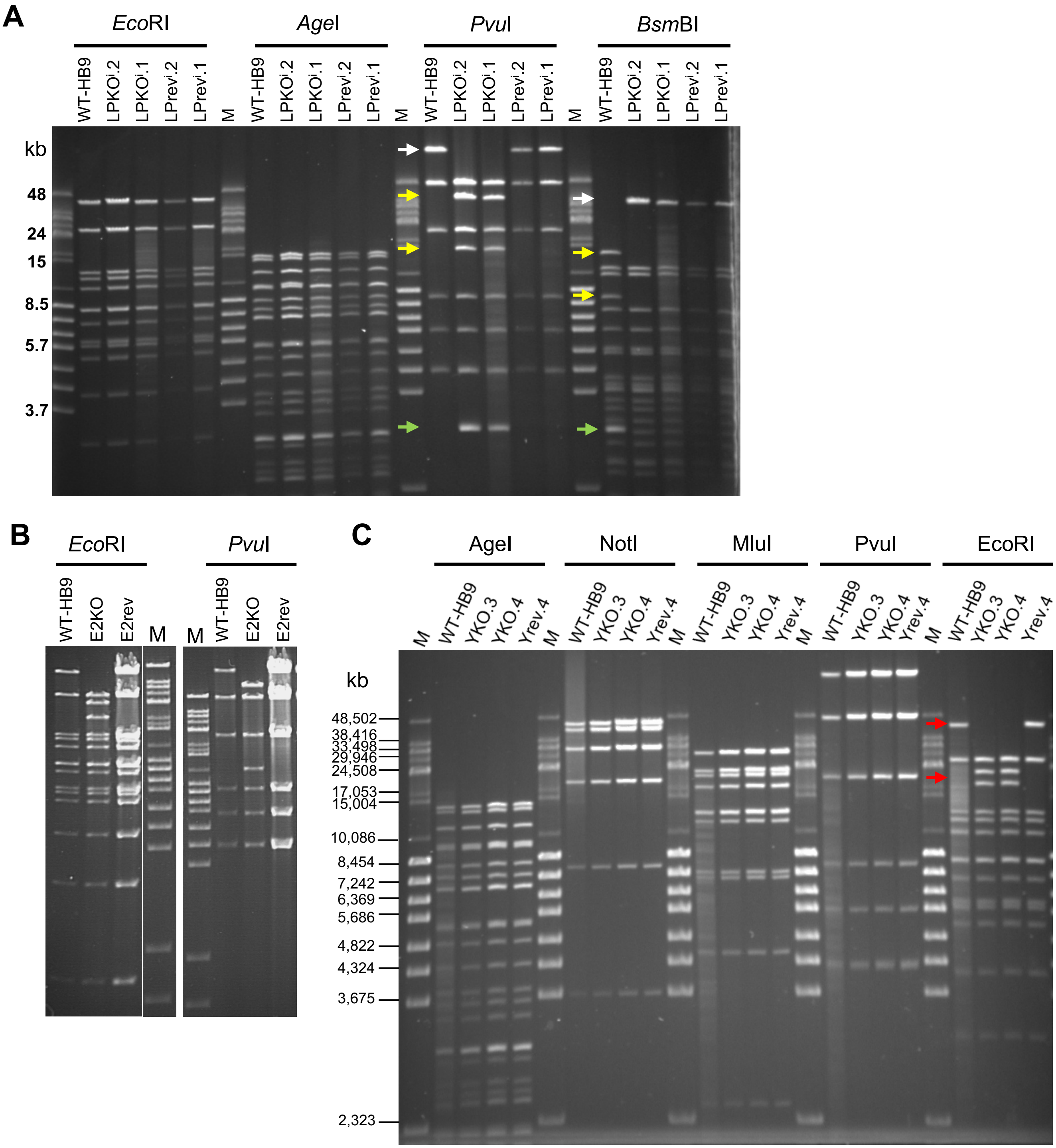

S2 Figure. Pulsed field gel analysis of recombinant EBVs. Analyses show the diagnostic digests for the construction of: **A.** LPKO^{i} and its revertand LPrev^{i}; **B.** E2KO and E2rev; **C.** YKO and Yrev. The size standard marker (M) is a 1:1 mixture of BstEII-lambda and Lambda mono-cut marker (NEB). **A.** Recombinant LPKO^{i} and LPrev^{i} viruses are identical, including all containing 6.6 IR1 repeats, other than bands altered by the inserted PvuI restriction site or removal of BsmBI. Digestion at these sites results in conversion of the IR1 band (white arrow) into the 3kb IR1 repeat unit (green arrow) and the Cp and Y bands flanking the repeat (yellow arrows). **B.** Size changes in E2KO result from introduction of EcoRI and PvuI restriction sites. **C.** YKO mutation produces a 140bp reduction in band size that is too small to detect in these digests, and an introduced EcoRI restriction site that causes a more easily observed change (red arrows). All other bands are unchanged, demonstrating the integrity of the genome outside the intended mutations.
