# Supplementary material for: Epstein-Barr virus nuclear antigen EBNA-LP is essential for transforming naïve B cells, and facilitates recruitment of transcription factors to the viral genome
Source: PLoS Pathog. 2018 Feb 20;14(2):e1006890. doi: 10.1371/journal.ppat.1006890 (PMC5834210; doi:10.1371/journal.ppat.1006890)
Supplement: S3 Fig — A. To test whether the splicing of EBNA transcripts had been affected by the changes inserted into the viruses, PCRs were conducted between the C1 and W0 exons (upstream) and the YH exon downstream to compare the transcripts produced by wild-type EBV and the LPKOi, LPrevi, and YKO EBVs. B. Western blotting of EBV protein levels in BL31 cells stably infected with the various recombinant viruses. A and B suffixes indicate independent BL31 cell lines produced from the same virus. (PDF) [file ppat.1006890.s003.pdf]

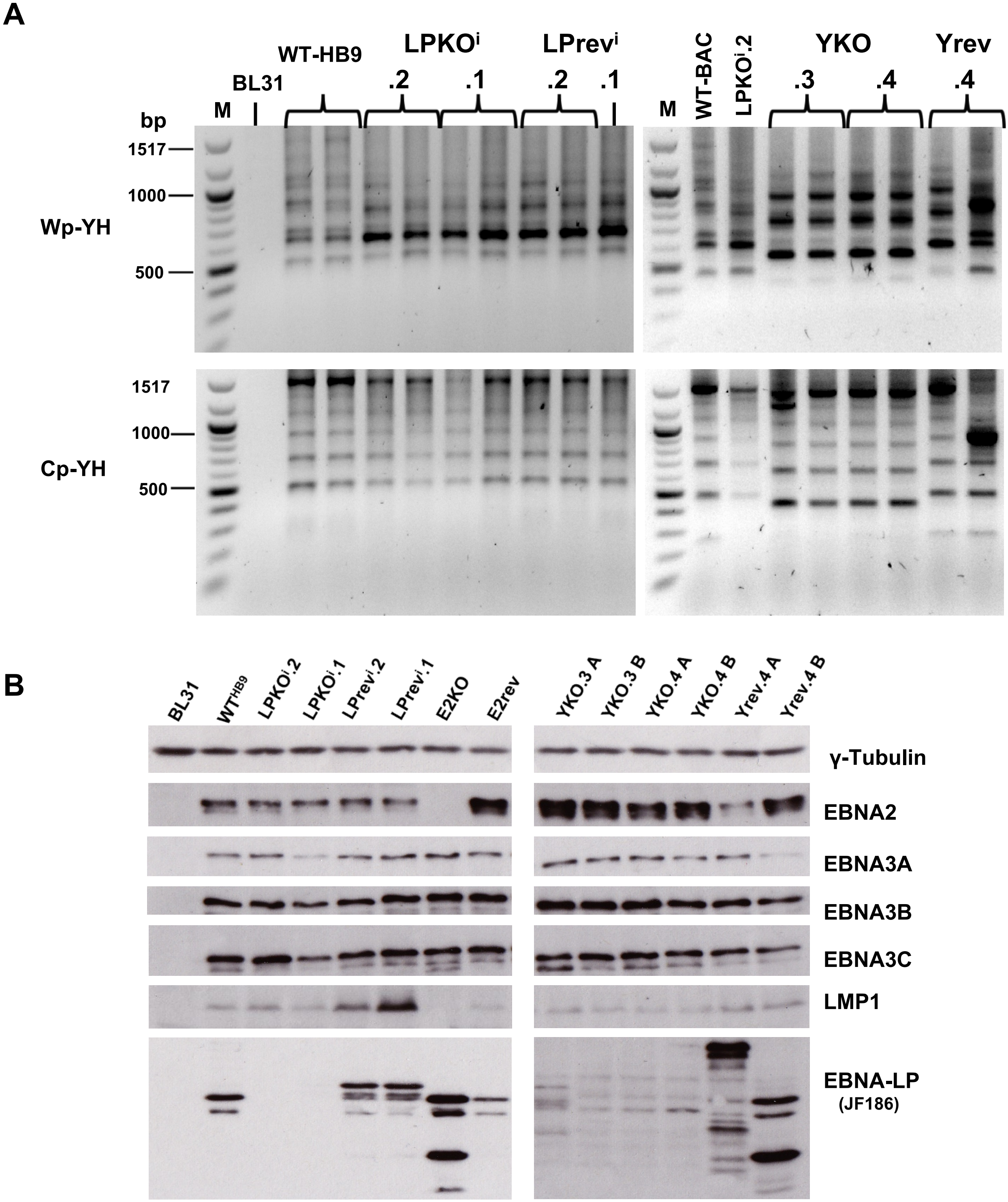

S3 Figure. recombinant EBV validation in BL31 cells. A. To test whether the splicing of EBNA transcripts had been affected by the changes inserted into the viruses, PCRs were conducted between the C1 and W0 exons (upstream) and the YH exon downstream to compare the transcripts produced by wild-type EBV and the LPKO^{i}, LPrev^{i}, and YKO EBVs. **B.** Western blotting of EBV protein levels in BL31 cells stably infected with the various recombinant viruses. A and B suffixes indicate independent BL31 cell lines produced from the same virus.
