# Supplementary material for: Epstein-Barr virus nuclear antigen EBNA-LP is essential for transforming naïve B cells, and facilitates recruitment of transcription factors to the viral genome
Source: PLoS Pathog. 2018 Feb 20;14(2):e1006890. doi: 10.1371/journal.ppat.1006890 (PMC5834210; doi:10.1371/journal.ppat.1006890)
Supplement: S4 Fig — Various western blots for EBV proteins in cell lines infected with EBNA2 knockouts and revertants. Each lane is identified by the virus recombinant, above the identifier of the 293 cell virus producer line, and bottom is the BL31 cell line ID. Each lane therefore represents an independent cell line. Note that BL31-E2KO-GK is a cell line generated using a different EBNA2-knockout EBV, produced by Gemma Kelly and Alan Rickinson [34]. (PDF) [file ppat.1006890.s004.pdf]

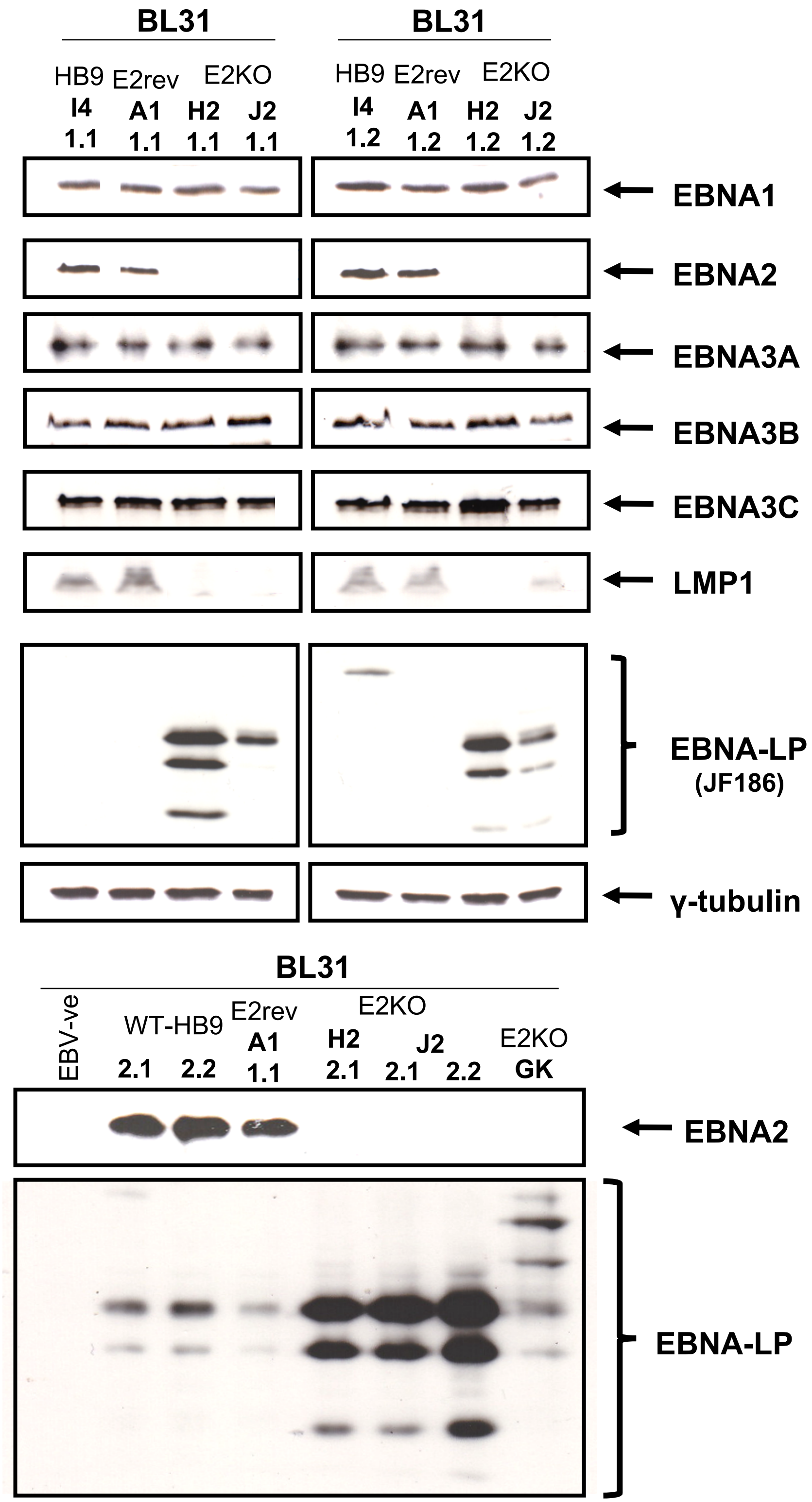

S4 Figure. Western blot validation of EBNA2 knockouts in BL31 cells. Various western blots for EBV proteins in cell lines infected with EBNA2 knockouts and revertants. Each lane is identified by the virus recombinant, above the identifier of the 293 cell virus producer line, and bottom is the BL31 cell line ID. Each lane therefore represents an independent cell line. Note that BL31-E2KO-GK is cell line generated using a different EBNA2-knockout EBV, produced by Gemma Kelly and Alan Rickinson [34].
