# Supplementary material for: Epstein-Barr virus nuclear antigen EBNA-LP is essential for transforming naïve B cells, and facilitates recruitment of transcription factors to the viral genome
Source: PLoS Pathog. 2018 Feb 20;14(2):e1006890. doi: 10.1371/journal.ppat.1006890 (PMC5834210; doi:10.1371/journal.ppat.1006890)
Supplement: S5 Fig — Antibodies used to label proteins are shown as indicated. EBV-infected cells were reproducibly seen associated with pericellular foci that were labelled by the anti-mouse secondary antibody alone. These are indicated by purple arrows. Yellow arrows indicate an apparently nucleolar accumulation of the truncated EBNA-LP in YKO infections. The red single channel image in YKO has been brightened to improve visualisation of the faint EBNA-LP signal. Other channels use the same brightness across the experiment. Note the extremely intense staining of EBNA-LP in E2KO infected cells. (PDF) [file ppat.1006890.s005.pdf]

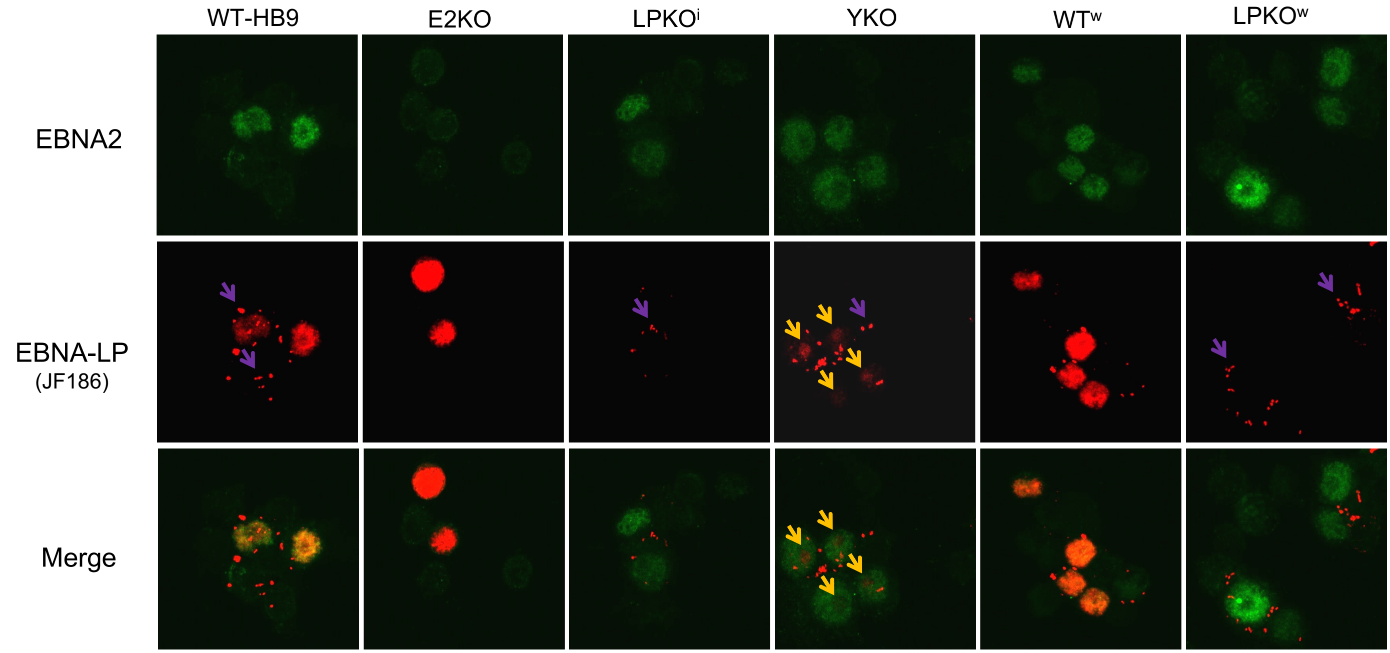

S5 Figure. Immunofluorescence analysis of EBNA2 and EBNA-LP expression after infection of primary B cells 48 hours post infection. Antibodies used to label proteins are shown as indicated. EBV-infected cells were reproducibly seen associated with pericellular foci that were labelled by the anti-mouse secondary antibody alone. These are indicated by purple arrows. Yellow arrows indicate an apparently nucleolar accumulation of the truncated EBNA-LP in YKO infections. The red single channel image in YKO has been brightened to improve visualisation of the faint EBNA-LP signal. Other channels use the same brightness across the experiment. Note the extremely intense staining of EBNA-LP in E2KO infected cells.
