# Supplementary material for: Epstein-Barr virus nuclear antigen EBNA-LP is essential for transforming naïve B cells, and facilitates recruitment of transcription factors to the viral genome
Source: PLoS Pathog. 2018 Feb 20;14(2):e1006890. doi: 10.1371/journal.ppat.1006890 (PMC5834210; doi:10.1371/journal.ppat.1006890)
Supplement: S6 Fig — Photographs of the accumulation of transformed cells after infection of CD19-purified B cells by various EBV strains, taken on days 2–20 post infection as indicated. Activated cells form clusters that then proliferate to differing extents. (PDF) [file ppat.1006890.s006.pdf]

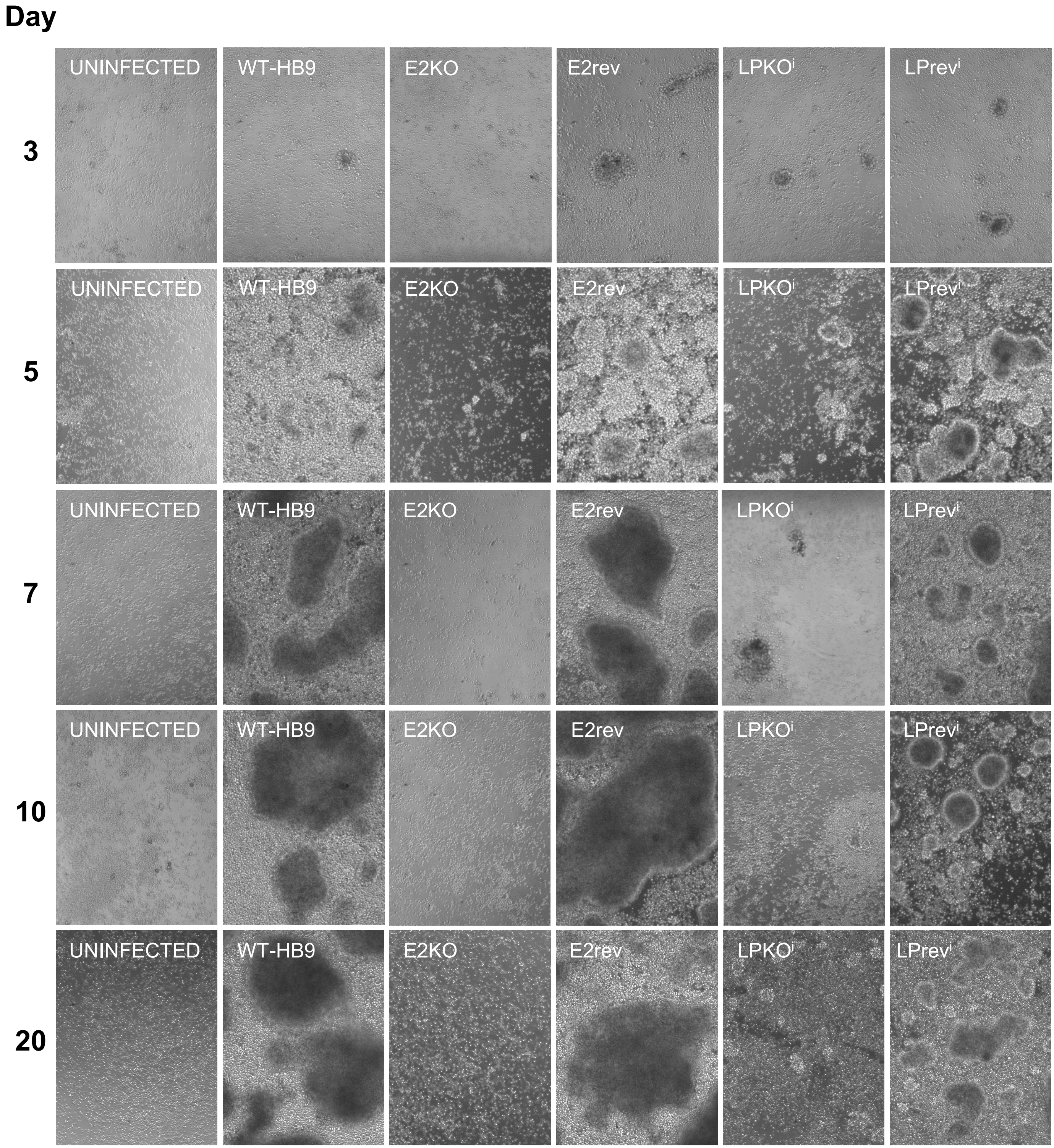

S6 Figure. Transformation of B cells by recombinant viruses. Photographs of the accumulation of transformed cells after infection of CD19-purified B cells by various EBV strains, taken on days 2-20 post infection as indicated. Activated cells form clusters that then proliferate to differing extents.
