# Supplementary material for: Epstein-Barr virus nuclear antigen EBNA-LP is essential for transforming naïve B cells, and facilitates recruitment of transcription factors to the viral genome
Source: PLoS Pathog. 2018 Feb 20;14(2):e1006890. doi: 10.1371/journal.ppat.1006890 (PMC5834210; doi:10.1371/journal.ppat.1006890)
Supplement: S7 Fig — Western blots of proteins from LCLs grown out from recombinant EBV infections. The virus used for the outgrowth is indicated. Initial phase of the outgrowth of cells was either performed on irradiated MRC5 feeder cells (F) or without feeder cells (N). The epitope in EBNA-LP recognised by the JF186 antibody exists in B95-8 but is missing from most virus strains. Antibody 4D3 recognises all known EBNA-LP variants. (PDF) [file ppat.1006890.s007.pdf]

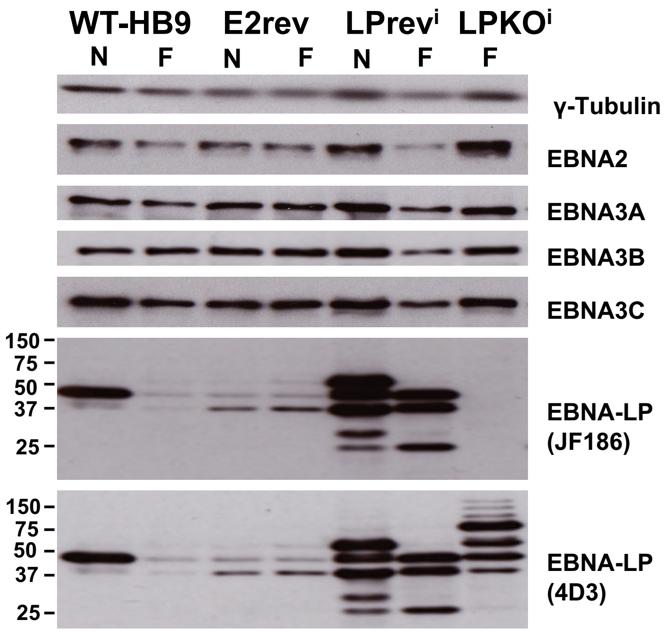

S7 Figure. Western Blot characterisation of LPKO^i, LPrev^i and YKO-established LCLs. Western blots of proteins from LCLs grown out from recombinant EBV infections. The virus used for the outgrowth is indicated. Initial phase of the outgrowth of cells was either performed on irradiated MRC5 feeder cells (F) or without feeder cells (N). The epitope in EBNA-LP recognised by the JF186 antibody exists in B95-8 but is missing from most virus strains. Antibody 4D3 recognises all known EBNA-LP variants.
