# Supplementary material for: Epstein-Barr virus nuclear antigen EBNA-LP is essential for transforming naïve B cells, and facilitates recruitment of transcription factors to the viral genome
Source: PLoS Pathog. 2018 Feb 20;14(2):e1006890. doi: 10.1371/journal.ppat.1006890 (PMC5834210; doi:10.1371/journal.ppat.1006890)
Supplement: S9 Fig — A. Schematic representation of the Gibson assembly strategy used to generate LPKOw and WTw. Grey boxes represent the BamW fragment and white boxes the flanks of the repeat as described in S1 Fig. Red and orange arrows indicate the sequences either side of the BamHI restriction site within IR1. These arrows are the homology regions whose overlap drives the Gibson assembly of overlapping fragments as indicated in the lower part of the figure, which shows the assembly of wild-type BamW fragments into the IR1 used to generate WTw. To generate LPKOw, the mutated W exons were cloned into each of the five plasmids indicated, and the assembly performed in the same way. B. Pulsed field gel analysis of the recombinant WTw and LPKOw viruses compared to the parental WTHB9. The PvuI digest shows the presence of the knockout-specific mutation in EBNA-LP (yellow arrows), releasing multiple copies of the 3kb IR1 repeat unit (white arrow), as compared to the parental BAC (WTHB9) and WTw. The other digests show the overall integrity of the rest of the virus genome. (PDF) [file ppat.1006890.s009.pdf]

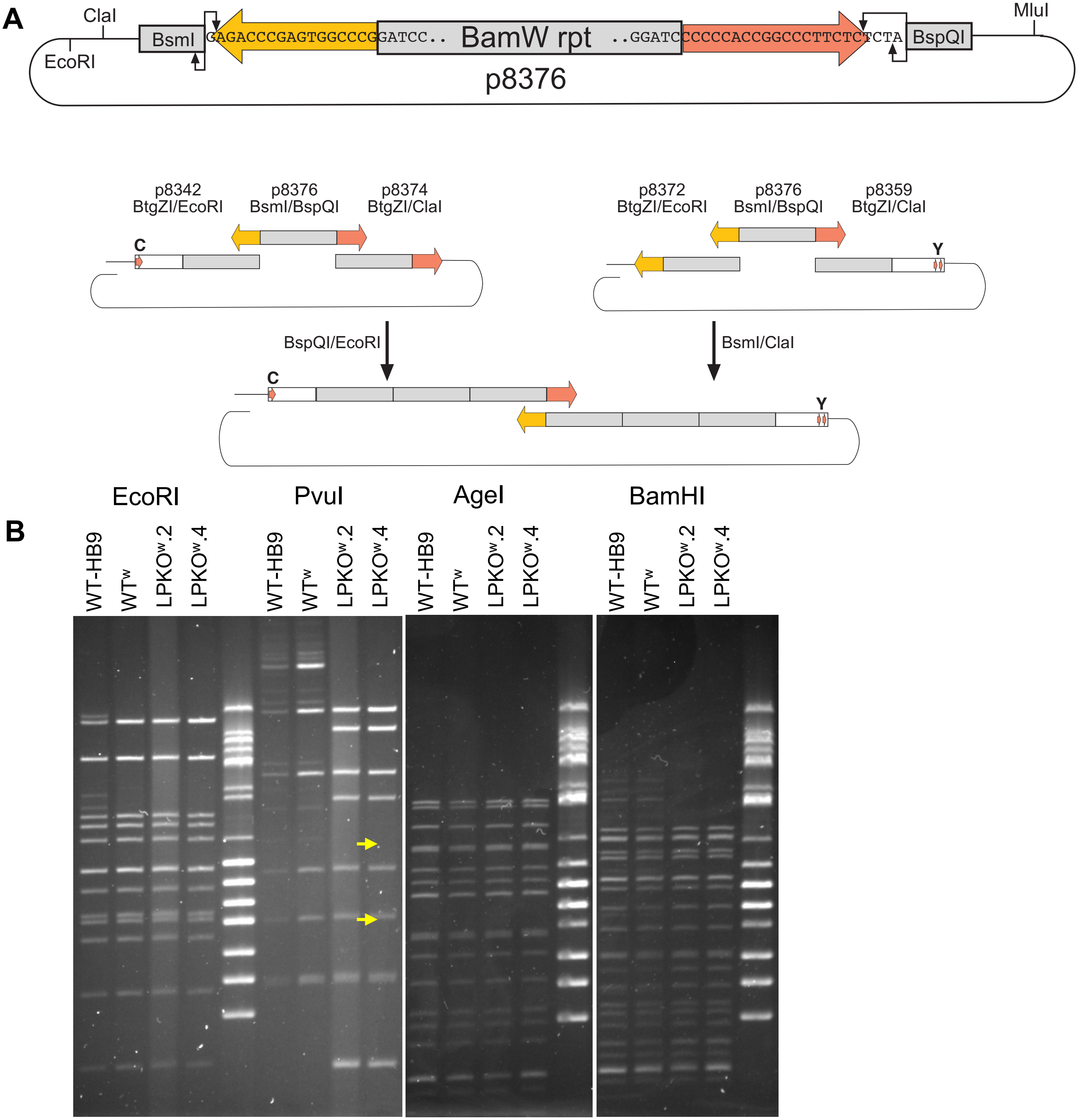

S9 Figure. Generation of recombinant viruses containing an IR1 repeat produced by Gibson assembly. A. Schematic representation of the Gibson assembly strategy used to generate LPKO^{w} and WT^{w}. Grey boxes represent the BamW fragment and white boxes the flanks of the repeat as described in S1 Figure. Red and orange arrows indicate the sequences either side of the BamHI restriction site within IR1. These arrows are the homology regions whose overlap drives the Gibson assembly of overlapping fragments as indicated in the lower part of the figure, which shows the assembly of wild-type BamW fragments into the IR1 used to generate WT^{w}. To generate LPKO^{w}, the mutated W exons were cloned into each of the five plasmids indicated, and the assembly performed in the same way. **B.** Pulsed field gel analysis of the recombinant WT^{w} and LPKO^{w} viruses compared to the parental WT^{HB9}. The PvuI digest shows the presence of the knockout-specific mutation in EBNA-LP (yellow arrows), releasing multiple copies of the 3kb IR1 repeat unit (white arrow), as compared to the parental BAC (WT^{HB9}) and WT^{w}. The other digests show the overall integrity of the rest of the virus genome.
