# Supplementary material for: Epstein-Barr virus nuclear antigen EBNA-LP is essential for transforming naïve B cells, and facilitates recruitment of transcription factors to the viral genome
Source: PLoS Pathog. 2018 Feb 20;14(2):e1006890. doi: 10.1371/journal.ppat.1006890 (PMC5834210; doi:10.1371/journal.ppat.1006890)
Supplement: S10 Fig — Flow cytometry plots from live CD20-positive cells harvested either 4, 11 or 15 days after infection of adult B cells stained with CellTrace violet prior to infection. Degree of dilution of the violet signal is indicated on the x-axis, indicating number of cell divisions. Proliferation of infected cells was measured by dilution of CellTrace violet. Data for day 8 are found in Fig 3A. (PDF) [file ppat.1006890.s010.pdf]

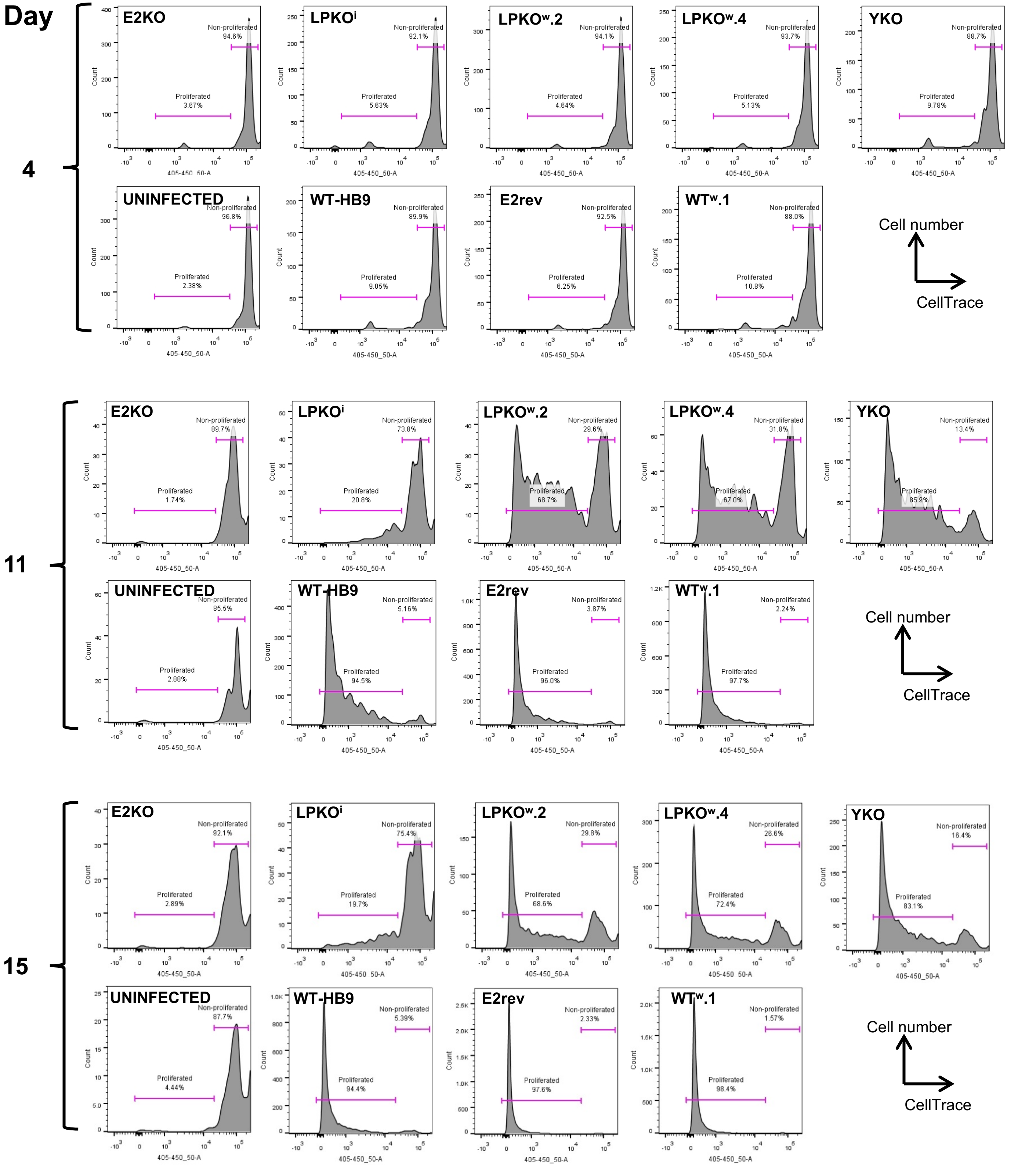

S10 Figure. Proliferation of cell lines at various time points. Flow cytometry plots from live CD20-positive cells harvested either 4, 11 or 15 days after infection of adult B cells stained with CellTrace violet prior to infection. Degree of dilution of the violet signal is indicated on the x-axis, indicating number of cell divisions. Proliferation of infected cells was measured by dilution of CellTrace violet. Data for day 8 are found in Fig 3A.
