# Supplementary material for: Epstein-Barr virus nuclear antigen EBNA-LP is essential for transforming naïve B cells, and facilitates recruitment of transcription factors to the viral genome
Source: PLoS Pathog. 2018 Feb 20;14(2):e1006890. doi: 10.1371/journal.ppat.1006890 (PMC5834210; doi:10.1371/journal.ppat.1006890)
Supplement: S11 Fig — Example flow cytometry plot of magnetically purified B cells (CD19+ve) stained with CD27 and IgD. Coloured boxes show the position of the gates used to separate the four subsets. IgD-/CD27- B cells (Magenta) are not a functionally defined subset (or could be non-B cells), so have not been named. (PDF) [file ppat.1006890.s011.pdf]

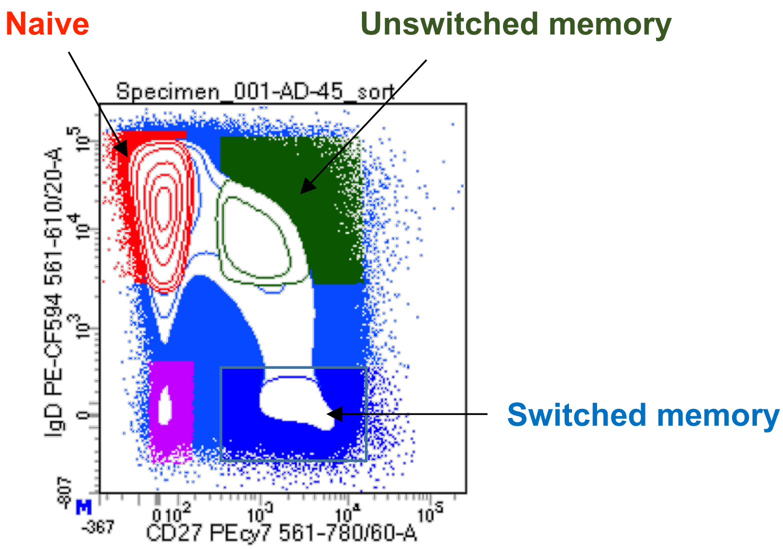

S11 Figure. Gating strategy used to define B cell subsets. Example flow cytometry plot of magnetically purified B cells (CD19+ve) stained with CD27 and IgD. Coloured boxes show the position of the gates used to to separate the four subsets. IgD-/CD27- B cells (Magenta) are not a functionally defined subset (or could be non-B cells), so have not been named.
