# Supplementary material for: Epstein-Barr virus nuclear antigen EBNA-LP is essential for transforming naïve B cells, and facilitates recruitment of transcription factors to the viral genome
Source: PLoS Pathog. 2018 Feb 20;14(2):e1006890. doi: 10.1371/journal.ppat.1006890 (PMC5834210; doi:10.1371/journal.ppat.1006890)
Supplement: S12 Fig — Plots show the IgD (y-axis) and CD27 (x-axis) status for the pre and post-sort populations from six donors, with donor ID shown in the top of each red box. For each donor, the top two plots show the pre-sort population (total events left; smaller number of events right). The lower plots for each show the identity of each sorted population with the percentage purity shown as magenta numbers. (PDF) [file ppat.1006890.s012.pdf]

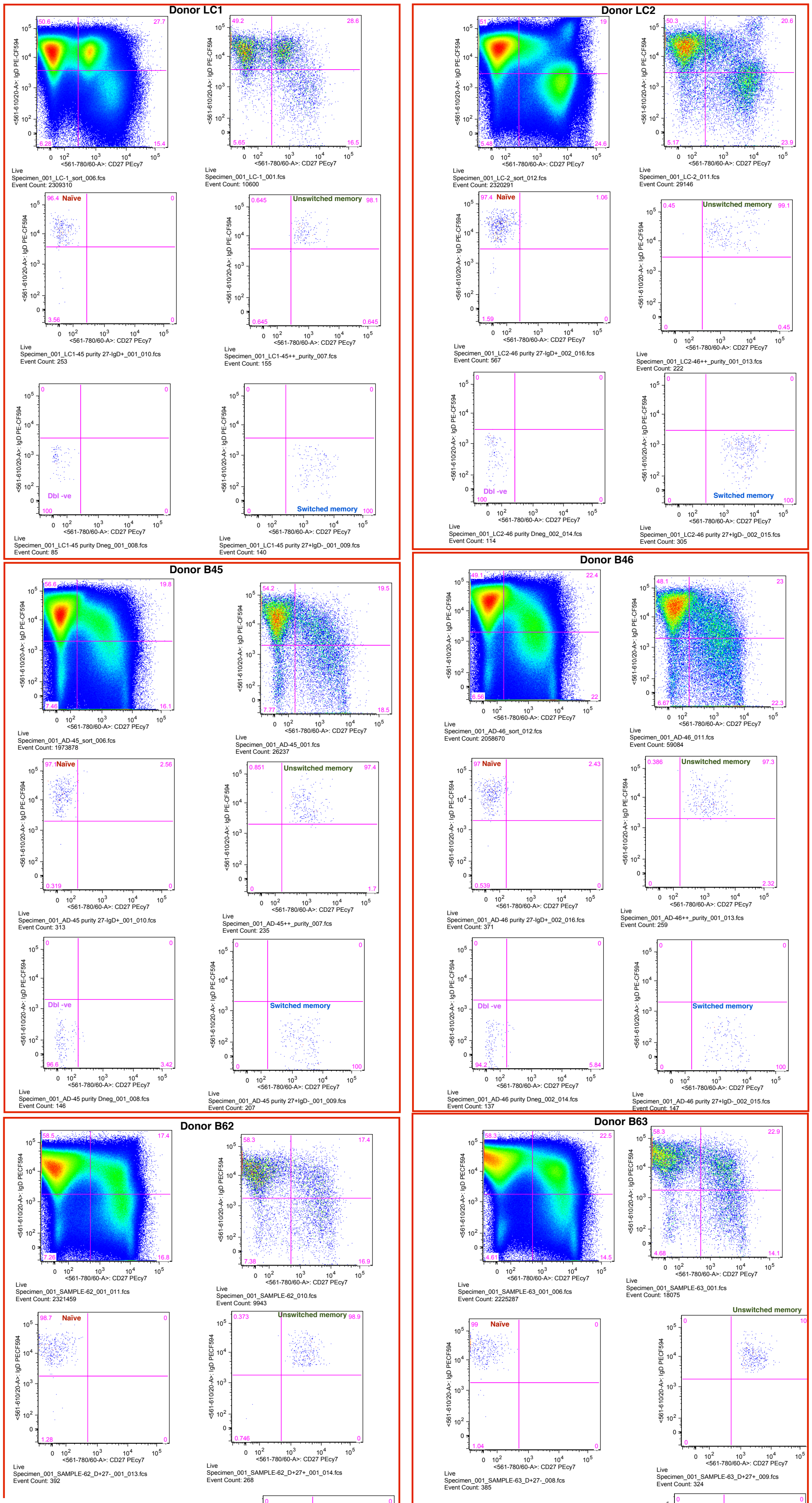

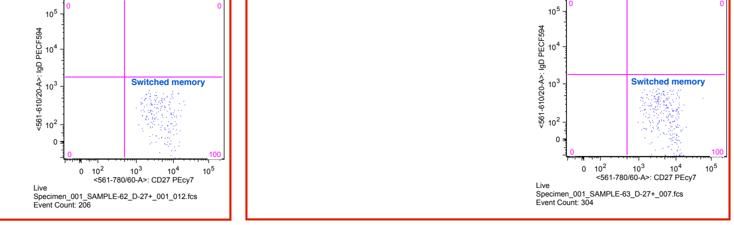

S12 Figure. FACS sorter and purity plots.

Plots show the IgD (y-axis) and CD27 (x-axis) status for the pre and post-sort populations from six donors, with donor ID shown in the top of each red box. For each donor, the top two plots show the pre-sort population (total events left; smaller number of events right). The lower plots for each show the identity of each sorted population with the percentage purity shown as magenta numbers.
