# Supplementary material for: Epstein-Barr virus nuclear antigen EBNA-LP is essential for transforming naïve B cells, and facilitates recruitment of transcription factors to the viral genome
Source: PLoS Pathog. 2018 Feb 20;14(2):e1006890. doi: 10.1371/journal.ppat.1006890 (PMC5834210; doi:10.1371/journal.ppat.1006890)
Supplement: S13 Fig — A. Flow cytometry plots show the CD27 (x-axis) and IgD status (y-axis) of LCLs established with different viruses (top labels) in different B cell subsets (labels left) from donor B62. Numbers in the plots show the percentage of cells in each quadrant, used to plot Fig 5B and 5C. Naïve B cell-derived LCLs were all assayed on the same day, 46 days post infection. B. Similar analysis of LCLs established from donors LC1 and LC2 (where memory-derived LCLs were from a mixture of switched and unswitched memory B cells). (PDF) [file ppat.1006890.s013.pdf]

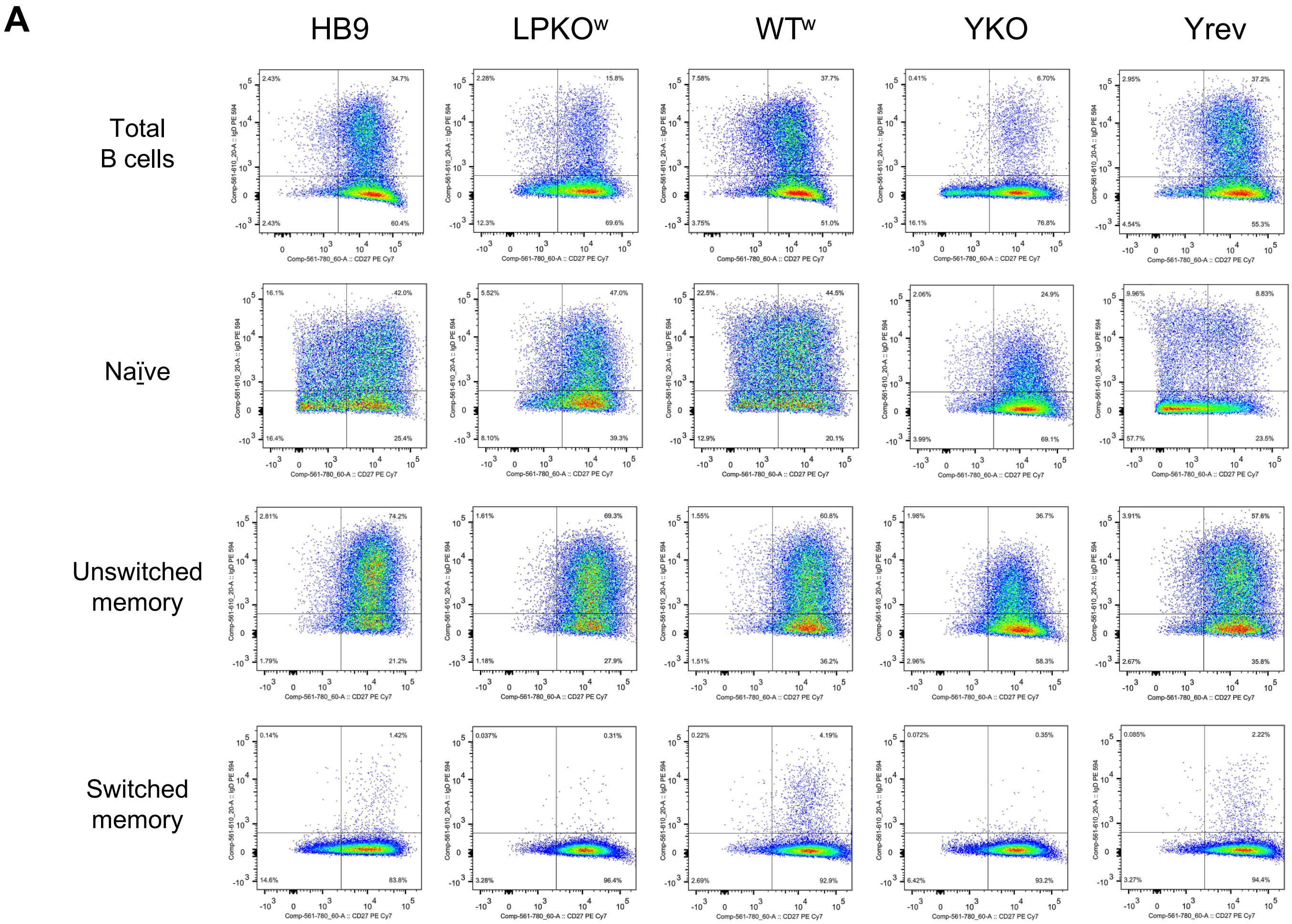

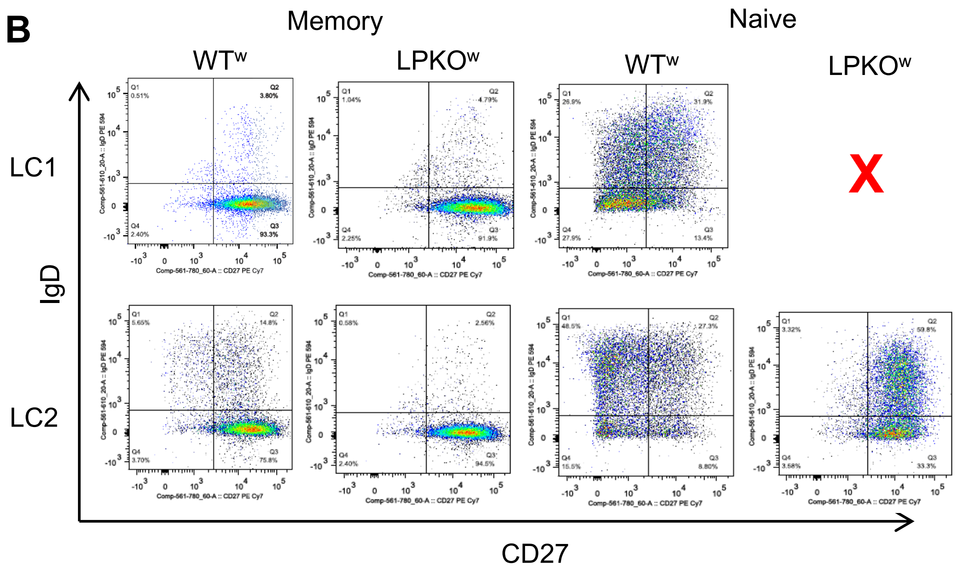

S13 Figure. EBNA-LP-null LCLs only establish with a memory B cell-like phenotype. A. Flow cytometry plots show the CD27 (x-axis) and IgD status (y-axis) of LCLs established with different viruses (top labels) in different B cell subsets (labels left) from donor B62. Numbers in the plots show the percentage of cells in each quadrant, used to plot Fig 5B/C. Naïve B cell-derived LCLs were all assayed on the same day, 46 days post infection. **B.** Similar analysis of LCLs established from donors LC1 and LC2 (where memory-derived LCLs were from a mixture of switched and unswitched memory B cells).
