# Supplementary material for: Epstein-Barr virus nuclear antigen EBNA-LP is essential for transforming naïve B cells, and facilitates recruitment of transcription factors to the viral genome
Source: PLoS Pathog. 2018 Feb 20;14(2):e1006890. doi: 10.1371/journal.ppat.1006890 (PMC5834210; doi:10.1371/journal.ppat.1006890)
Supplement: S14 Fig — Time course of transcript levels based on Taqman qPCR assays spanning: A. Exon W0 to W1 to indicate activity of the Wp promoters; B. Exon C1/2 to W1 to measure Cp promoter activity; and C. spanning exons 5 and 6 of LMP2 that measures LMP2 transcripts originating from LMP2A exon 1, LMP2B exon 1, and terminal repeats that should constitute all LMP2 transcripts. (PDF) [file ppat.1006890.s014.pdf]

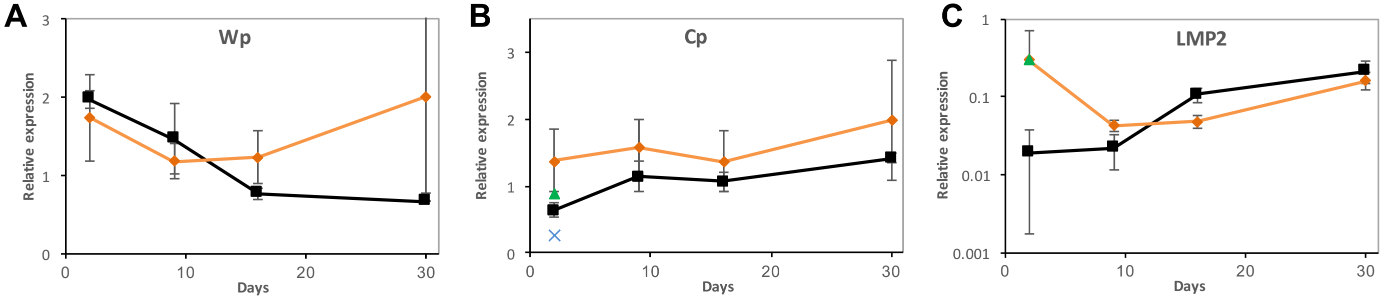

S14 Figure. QPCR analysis of Cp, Wp and total LMP2 transcription. Time course of transcript levels based on Taqman qPCR assays spanning: **A**. Exon W0 to W1 to indicate activity of the Wp promoters; **B**. Exon C1/2 to W1 to measure Cp promoter activity; and **C**. spanning exons 5 and 6 of LMP2 that measures LMP2 transcripts originating from LMP2A exon 1, LMP2B exon 1, and terminal repeats that should constitute all LMP2 transcripts.
