# Supplementary material for: Epstein-Barr virus nuclear antigen EBNA-LP is essential for transforming naïve B cells, and facilitates recruitment of transcription factors to the viral genome
Source: PLoS Pathog. 2018 Feb 20;14(2):e1006890. doi: 10.1371/journal.ppat.1006890 (PMC5834210; doi:10.1371/journal.ppat.1006890)
Supplement: S1 Fig — A. Intronic mutation introduced to remove a BsmBI restriction site in the intron to allow this cloning method to be used. B. Method for the construction of LPKOi and LPrevi viruses. Type IIS restriction enzyme sites used to assemble repeat arrays were designed to cut at the same site as BamHI in a pBR322-based plasmid. The BamHI sub-fragment (BamW) was subcloned into the BamHI site in the orientation indicated. The internal BsmBI restriction site that was mutated to allow this construction method is outlined by a green box. Other features of the IR1 repeat are indicated. C. Cloning strategy for the assembly of LPKOi is shown. LPrevi assembly followed an equivalent series of cloning steps. Grey boxes indicate BamW fragments, while white boxes indicate the SfiI/BamHI or BamHI/MluI regions at the edges of IR1 as shown in A above. Black box within BamW represents the mutation of EBNA-LP and the deleted BsmBI restriction site. Plasmid IDs are indicated. C and Y indicate the exons at the flanks of the targeting region. (PDF) [file ppat.1006890.s001.pdf]

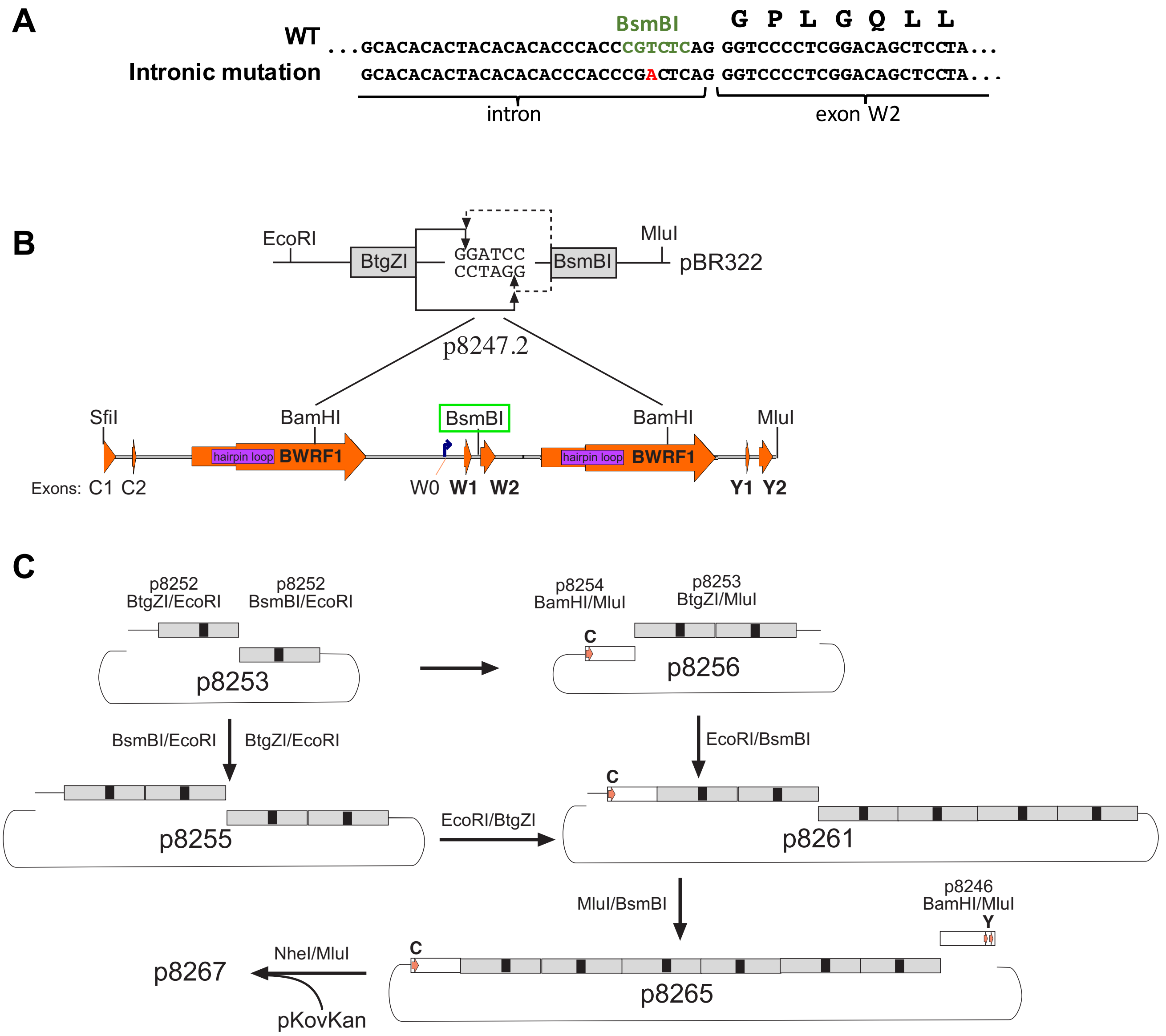

S1 Figure. Generation of viruses containing recombinant IR1 using type IIS restriction enzymes. A. Intronic mutation introduced to remove a BsmBI restriction site in the intron to allow this cloning method to be used. **B.** Method for the construction of LPKO[i] and LPrev[i] viruses. Type IIS restriction enzyme sites used to assemble repeat arrays were designed to cut at the same site as BamHI in a pBR322-based plasmid. The BamHI sub-fragment (BamW) was subcloned into the BamHI site in the orientation indicated. The internal BsmBI restriction site that was mutated to allow this construction method is outlined by a green box. Other features of the IR1 repeat are indicated. **C.** Cloning strategy for the assembly of LPKO[i] is shown. LPrev[i] assembly followed an equivalent series of cloning steps. Grey boxes indicate BamW fragments, while white boxes indicate the SfiI/BamHI or BamHI/MluI regions at the edges of IR1 as shown in A above. Black box within BamW represents the mutation of EBNA-LP and the deleted BsmBI restriction site. Plasmid IDs are indicated. C and Y indicate the exons at the flanks of the targeting region.
